# Self-Immolative Electrochemical Redox Substrates: Emerging Artificial Receptors in Sensing and Biosensing

**DOI:** 10.1021/acsmeasuresciau.3c00057

**Published:** 2024-01-18

**Authors:** Kesavan Manibalan, Ponnusamy Arul, Hsin-Jay Wu, Sheng-Tung Huang, Veerappan Mani

**Affiliations:** † Department of Materials Science and Engineering, National Yang Ming Chiao Tung University, Hsinchu 30010, Taiwan; ‡ Institute of Biochemical and Biomedical Engineering, Department of Chemical Engineering and Biotechnology, National Taipei University of Technology, Taipei 10608, Taiwan (ROC); § Advanced Membranes and Porous Materials Center (AMPMC), Computer, Electrical and Mathematical Science and Engineering Division, King Abdullah University of Science and Technology (KAUST), Thuwal 23955-6900, Saudi Arabia; ¶ High-Value Biomaterials Research and Commercialization Center, National Taipei University of Technology, No. 1, Sec. 3, Zhongxiao E. Rd., Taipei 10608, Taiwan (ROC)

**Keywords:** artificial receptors, ratiometric sensors, electrochemical sensors and biosensors, electrochemical mediators, self-immolative sensors, galactosidase, alkaline phosphatase, neuraminidase, hydrogen peroxide, organic electrochemistry

## Abstract

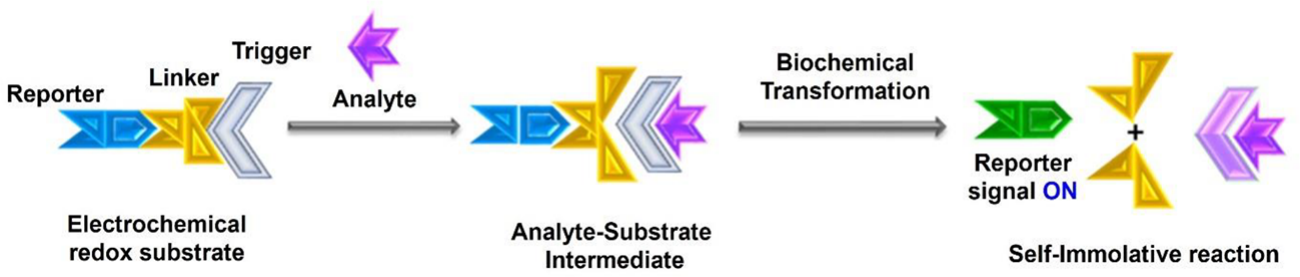

The development of artificial receptors has great significance in measurement science and technology. The need for a robust version of natural receptors is getting increased attention because the cost of natural receptors is still high along with storage difficulties. Aptamers, imprinted polymers, and nanozymes are some of the matured artificial receptors in analytical chemistry. Recently, a new direction has been discovered by organic chemists, who can synthesize robust, activity-based, self-immolative organic molecules that have artificial receptor properties for the targeted analytes. Specifically designed trigger moieties implant selectivity and sensitivity. These latent electrochemical redox substrates are highly stable, mass-producible, inexpensive, and eco-friendly. Combining redox substrates with the merits of electrochemical techniques is a good opportunity to establish a new direction in artificial receptors. This Review provides an overview of electrochemical redox substrate design, anatomy, benefits, and biosensing potential. A proper understanding of *molecular design* can lead to the development of a library of novel self-immolative redox molecules that would have huge implications for measurement science and technology.

## 1 Introduction

The design and development of artificial receptors in an active field of research in sensors and biosensors.^1^ Aptamers, imprinted polymers, and nanozymes are some of the matured artificial receptors in analytical chemistry. Recently, a new direction has been discovered via synthesizing robust, activity-based, self-immolative organic molecules that have artificial receptor property for the targeted analytes. In the past years, a wide range of chromogenic organic probes or substrates have been developed for imaging enzyme activity in live cells and animal models.^2−4^ The self-immolative substrates are widely explored in fluorescent sensors.^5^ Although chromogenic and fluorogenic probes have solved the problem of monitoring the spatiotemporal distribution of enzymes, they have certain limitations. First, optical methods require transparent samples and requiring additional optical tools that are often expensive to convert the underlying nonreadable analyte–substrate chemical interaction into a readable digital signal. Second, optical methods have certain generic limitations such as low quantum yield, autoabsorption/autofluorescence, and requirement for bulky machines.^6^ On the contrary, electrochemical assays are known for their low cost, portability, affordability, convenience of use, and direct digital readouts without requiring external optical tools.^7,8^ Therefore, electrochemical redox substrates equivalent to chromogenic substrates are recently getting more attention. Electrochemical substrates have emerged as a new class of artificial receptors for sensing a variety of biomarkers including metabolites, enzymes, proteins, and metal ions. This type of invented materials can drive a rapid ejection of an electrochemical reporter and be detected via test electrolytes. While Shabat and Gnaim reported a review article covering fluorogenic organic probes, so far, there is no review article reported on electrochemical molecular probes.^9^

Electrochemical redox substrates/probes are chemical molecules composed of a trigger group attached to a linker, which is linked to a latent electrochemical redox reporter.^10,11^ They are bench stable, biomimetic chemical species and are self-immolative. The specific trigger groups acting as receptors are the recognition sites for the target analytes. When the designer-specific trigger group is spark off with a corresponding analyte, the substrate goes through a predesigned self-immolative transformation to eject out the unmasked electrochemical reporter, which can be electrochemically probed. The ejected reporter presents a voltammetry or amperometric signal that is linearly correlated with the concentration of the biomarkers. These “trigger–linker–reporter” sensing methods are simple, selective, sensitive, low-cost, and portable and have the potential for point-of-care assays with minimal sample requirements. These electrochemical substrates, in contrast to natural sensors, have stable chemical bonds between the components and no delicate biological components, allowing them to be stored at room temperature. Because of these advantages, there is an increasing interest among the analytical community to develop self-immolative electrochemical probes for sensors and biosensors. The self-immolative latent redox electrochemical substrates are of particular interest for the precise quantification of nonredox active biomarkers: thus, can help to elucidate the complex of biospecies identification and their redox biology.

The need for robust versions of antibodies is getting increasing attention, mainly due to the high production cost of antibodies. In this Review, we discuss self-immolative, activity-based electrochemical molecular switches for electrochemical sensing and biosensing applications. Such substrates are highly stable, mass-producible, inexpensive, and eco-friendly. We considered only those papers reporting stimuli-responsive electrochemical substrates with the general chemical anatomy of “reporter–linker–trigger”. This Review provides an overview of the design, synthesis, and biosensing potential of a new, emerging class of artificial receptors. The proper understanding of molecular design can lead to the development of a library of novel self-immolative redox molecules that would have huge implications for analytical science. In addition, it will have an impact on organic chemists and provide a guide for them on how to develop organic materials for biosensing applications.

## 2 Anatomy of Electrochemical Redox Substrates

**Figure 1:**
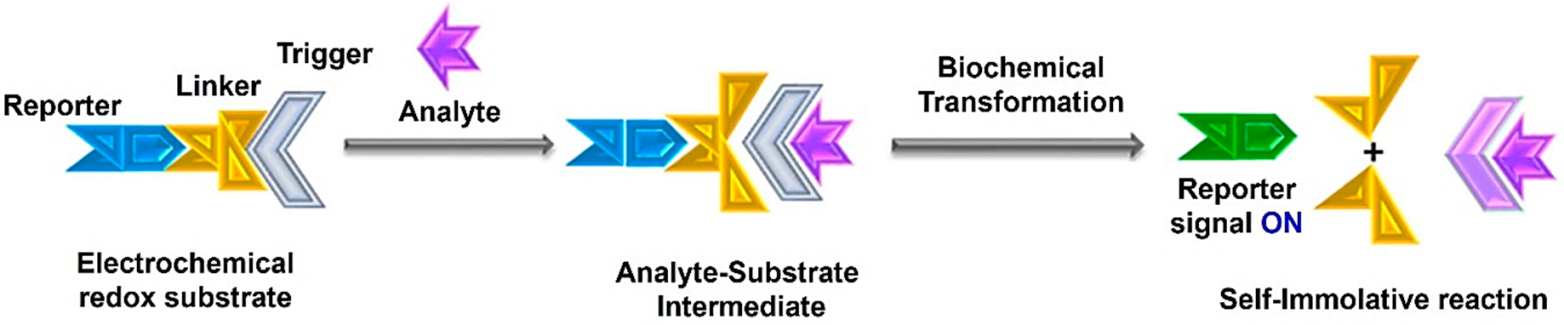
General sensing mechanism of activity-based self-immolative electrochemical redox substrates: Biomarker adduct, and disassembly pictography of an activity-based molecular switch.

The anatomy of a typical electrochemical substrate is sketched in Figure 1. In simple terms, electrochemical substrates are made up of three building blocks: (1) a reporter that produces a signal, (2) a trigger unit that specifically interacts with the analyte, and (3) a linker that bridges the reporter with the trigger. A proper understanding of their role is necessary to construct an electrochemical substrate successfully for a specific target biomarker. The following discussion provides a short introduction to these three building blocks of electrochemical substrates.

**Figure 2:**
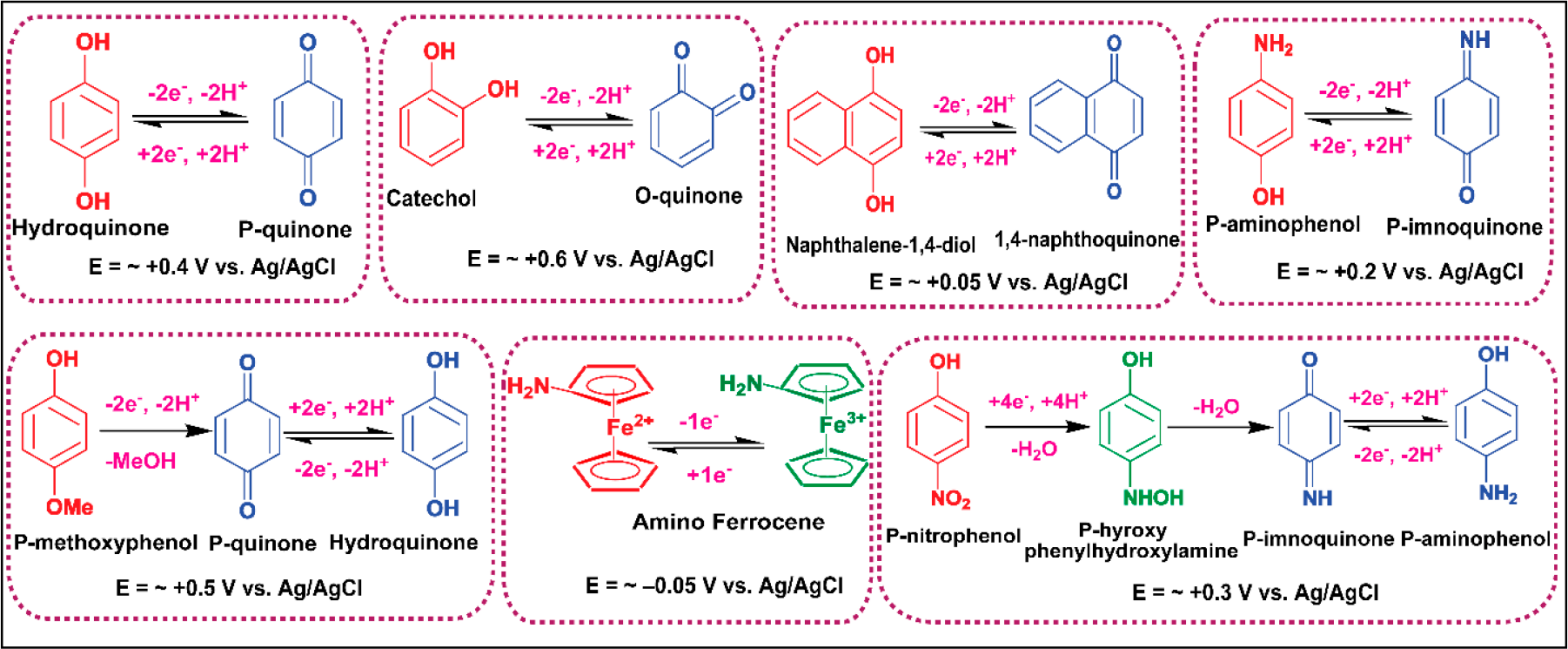
Chemical structures and redox reactions of most routinely used reporters to design electrochemical redox substrates.

**Figure 3:**
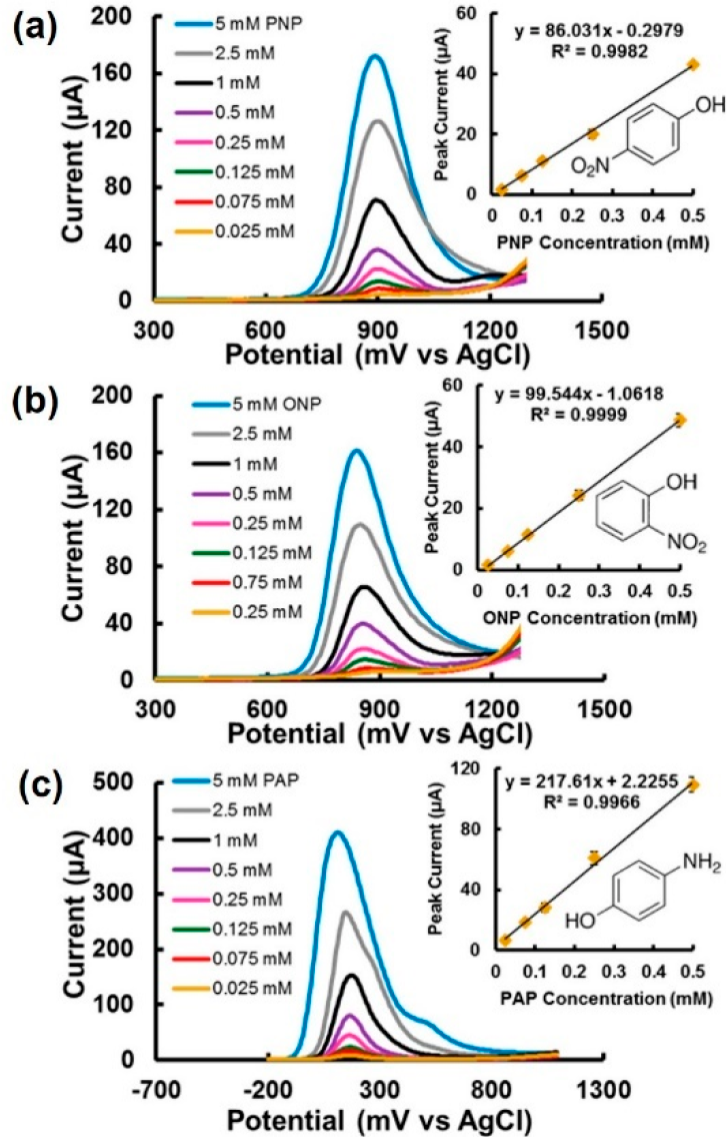
SWV signals of (a) *p*-nitrophenol (PNP), (b) *o*-nitrophenol (ONP), and (c) *p*-aminophenol (PAP) and their calibration plots in pH 6.5 phosphate buffered saline (PBS) buffer. The working electrode, reference electrode, and supporting electrolyte are stencil-printed transparency film-based carbon electrode, Ag/AgCl electrode, and PBS buffer (pH 6.50), respectively. Reprinted from ref (15). Copyright 2017 American Chemical Society.

Reporters are generally redox mediators that should have stable and sharp electrochemical signals in commonly used electrochemical cell systems. Their redox-active groups or fragments that are essential for showing redox signals are usually masked or protected with the help of linker moieties when preparing substrates. Therefore, the redox-active groups of the reporters should be flexible for chemical functionalization with linkers. For example, the two hydroxyl groups of hydroquinone can be easily functionalized with linkers such as silyl ethers^12^ or boronic esters.^13^ On the contrary, methylene blue which is the most preferred mediator in electrochemical sensors is not suitable for developed electrochemical redox substrates because its redox active groups are not easier to be protected or masked. The most widely used reporters are hydroquinone, naphthoquinone, catechol, *p*-nitrophenol, *p*-methoxyphenol, *p*-aminophenol, and aminoferrocene. The chemical structures, and redox reactions of the major reporters are sketched in Figure 2. The square wave voltammetry (SWV) signals of some of these reporters are given in Figure 3, which show stable and consistent electrochemical signals, making them more suitable to be used in ratiometric sensors. These reporter molecules have promising electrochemical properties and often show their redox signals at minimized overpotential regions on the electrochemical potential window. They can be easily functionalized or derivatized, water-soluble, and low-cost. As the analytical signal of the sensor is dictated by the reporter moiety, the solution properties of the reporter molecule should be considered to obtain a better sensing response. There will be a pH dependence if the redox behavior of the reporter molecule depends on the pH of the supporting solution.

**Figure 4:**
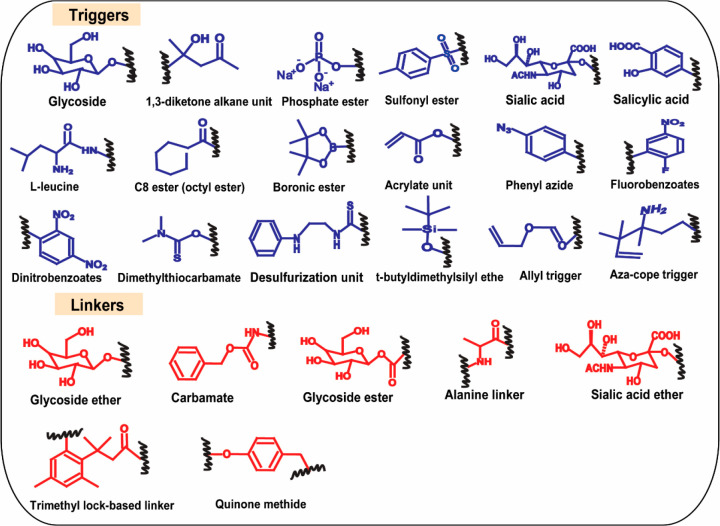
Triggers and linkers commonly used in electrochemical substrates.

Triggers play a crucial role in designing electrochemical redox reporters. As a rule, the trigger moiety should respond only to the specific target analyte for which the substrate is developed. In simple terms, triggers provide specificity and selectivity to the substrate molecules. The tailor-made specific trigger moieties are analyte recognition sites that serve as receptors. When such a trigger group is activated with a target analyte, the entire substrate molecule undergoes a planned self-immolative transformation to unmask the electrochemical reporter. Therefore, finding the right triggers is the first step in designing electrochemical redox substrates, this requires chemical reactivity knowledge from organic chemistry. Selectivity and anti-interference properties of the electrochemical redox substrates are majorly dictated by trigger moieties. The electrochemical reporters can retain their selectivity and anti-interference qualities as long as the triggers are not expelled by potentially interfering substances. Therefore, the linkage between triggers and linkers should consist of a stable bond that can only be broken by the target analyte and not by any of the other interfering substances. Some examples include: boronic ester-based triggers are specific for hydrogen peroxide (H_2_O_2_),^14^ silyl-based triggers are specific for fluoride detections,^12^ and azide-based trigger groups are specific for hydrogen sulfide.^10^ Designing triggers for enzymatic biomarkers requires a proper understanding of the biological functions of the enzymes and biochemical interactions with their target substrates. For example, β-galactosidopyranoside is the optimum trigger group for β-Galactosidase, which is derived by mimicking its biological substrate of gal β-d-galactoside residues and its function as a hydrolase. Similarly, triggers can be easily selected for other enzymes also, which are sketched in Figure 4.

Linkers are the backbone of electrochemical substrates that provide a proper connection between the trigger and the reporter. These linkers undergo rearrangement when substrates initiate interactions with their target analytes and are self-immolative in nature to eject the reporter as soon as possible. Some examples of linkers are ethers, esters, carbamates, etc. The type of the linker chains affects the elimination kinetics of the reporter, which plays a role in the elimination kinetics.^14^ Generally, a linker can be designed for the quickest release of the reporter to have a shorter incubation time with the target analyte, thus leading to a shorter analysis time. For instance, the p-benzyl carbamate linker showed faster elimination kinetics compared to the allyl carbamate linker for detecting H_2_O_2_.^14^ Finding effective linkers for achieving faster elimination kinetics is vital to increase sensor performance. Figure 4 illustrates the most widely used linkers for designing electrochemical substrates.

The selection of appropriate triggers, linkers, and reporters becomes easier once we understand their functions. Following selection, a relevant synthetic protocol for the synthesis of the substrate can be sketched. Generally, a synthetic approach with one step or fewer is favored. It should be noted that the study of electrochemical redox substrates necessitates an interdisciplinary approach involving knowledge from the fields of synthetic organic chemistry, analytical electrochemistry, and biotechnology.

## 3 Sensors for Non-oxidoreductase Enzymatic Biomarkers

Oxidoreductase biomarkers refer to several classes of biologically important molecules, such as proteins, enzymes, or small cell signaling molecules that oxidize or reduce to transmit the electronic response directly to the transducer device. For example, electrochemical evaluation of oxidoreductase species has been observed by direct electrical contact with an electrolyte-soluble redox partner such as cytochromes, hemoglobin, ferredoxin, vitamin B12, or plastocyanin that consists of redox-active sites or has an active functional group that gives a spontaneous response to the electrode via an electrocatalytic reaction.^16^ In this case, a biological molecule inherently consists of a redox active center that provides direct evaluation possibilities in biosensors.^17^ Unlike redox biomarkers, many electrochemically inactive biomarkers are widely available. When the nonredox active species come into direct contact with the transducer electrodes, natural estimation is impossible. In such cases, developing electrochemical reporters comprised of substrates/probes that enable the development of selective third-generation biosensors are interesting sensing technology. The combination of dynamic electrochemistry with organic latent redox substrates/probe techniques has now made it possible to characterize the nonoxidoreductase species via its electrode interfaces and electron transfer processes. This configuration has recently been shown to be useful in understanding the mechanisms of nonoxidoreductases and determining them accurately.^17^

The technology of activity-based enzymatic redox probes/substrates based on a self-immolative linker is crucial because enzyme activity varies dramatically with a small difference in the enzyme recognition unit produced in the substrates. Engineered substrates can facilitate precise enzyme-activated reactions or chemical transformations under biological conditions, resulting in new chemical products or enzyme-bound substrate molecules with distinct electrochemical properties.^18^ These new chemical species formed by biochemical changes respond to the change in electrochemical potential (voltage shift) and/or an increase/decrease in electrochemical current density (on or off).^10^ These reaction responses could be achieved by sensibly fabricating an enzyme recognition unit on the redox reporter through electrochemical reactions such as radical ion formation, molecular oxidation or reduction, and electron acceptor donor group exchange. In the case of radical ion formation, the substrate group bound to the reporter forms an enzyme-mediated cascade decomposition that releases the free reporter from the self-immolative linker and shows an electrochemical reaction by the type of reporter participating in the radial ion reaction (example: *p*-methoxyphenol). In electron acceptor donor group exchange, the reporter is connected to a substrate via an acceptor linker and converts to a donor-based free reporter to generate a potential-shifted electrochemical output when enzyme-mediated reactions occur (example: ferrocene derivatives, in this case, the probe generates its signal in the positive potential region and the reporter in the interference-free potential region). In the molecular oxidation or reduction case, the reporter is released by enzyme-mediated hydrolysis after a two-electron/two-proton transfer reaction, resulting in the generation or decrease of the reporter signal (e.g., *p*-nitrophenol, hydroquinone, etc.).

When developing activity-based electrochemical substrates, some additional criteria should be considered: (1) The reporter should have a specific voltage potential (interference-free region) to avoid other biological complexes. (2) The newly appearing electrochemical spectrum or electrochemical current density generated during biochemical conversion should have a sufficient signal-to-noise ratio to differentiate detection of the target analyte and increase sensitivity. (3) The probe should have sufficient aqueous solubility, cell permeability (for in situ biological studies), and low toxicity. (4) It should have high selectivity for specific enzymes. In addition, enzyme activities vary within the cell and in subcellular regions. Therefore, by using a cell-specific targeting moiety covalently linked to the electrochemical substrates probe skeleton, it is possible to study the continuous tracking of enzymes in tissues and subcellular compartments.

**Figure 5:**
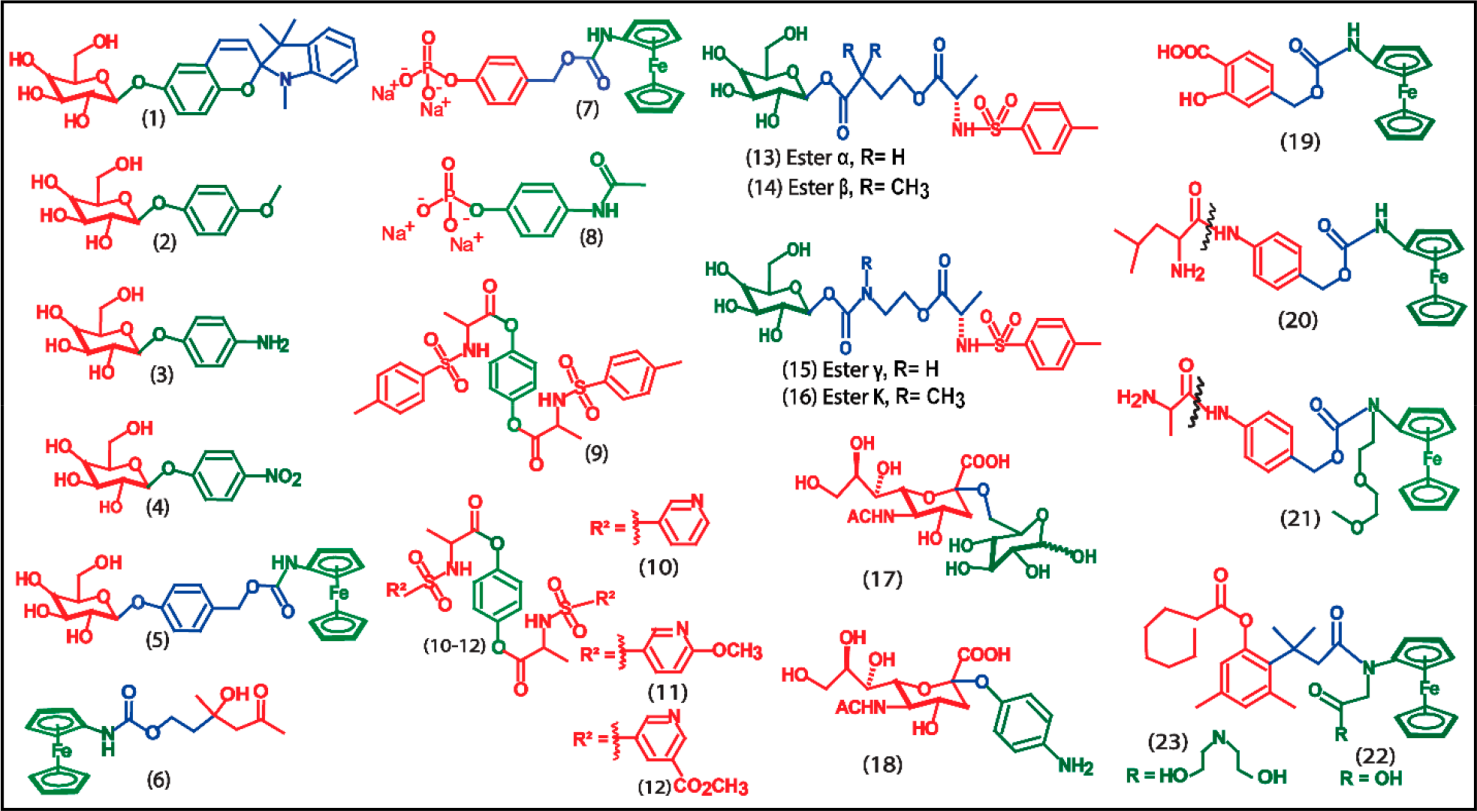
Structures of the electrochemical redox substrates (**1**–**22**). Spiropyran based electrochemical substrate, SP-β-gal (**1**), 4-methoxyphenyl-β-galactopyranoside, 4-MPGal (**2**), 4-aminophenyl-β-galactopyranoside, PAPG (**3**), *p*-nitrophenyl-β-galactopyranoside, PNPG (**4**), ferrocenylcarbamoylphenyl-β-d-galactosidase, FCPG **(5)**, ferrocene-aldol carbamate, FAC (**6**), ferrocenylphenyl phosphate (**7**), 4-Acetamidophenyl phosphate (**8**), 4-((tosyl-l-alanyl)oxy)phenyl tosyl-l-alaninate, TAPTA (**9**), water-soluble derivatives of TAPTA (**10**–**12**), glucosyl esters (**13**–**16**), sialic acid derivate, SG1 (**17**), *N*-acetyl-2-*O*-(4-aminophenyl)-neuraminic acid, AP-Neu5Ac (**18**), salicylic acid amino ferrocene, SAF (**19**), Leucine-benzyl ferrocene carbamate, Leu-FC (**20**), l-alanine recognition unit and an alkylated-ferrocenylamine, **AFA** (**21**), carboxyl aminoferrocene, Sal-CAF (**22**), and (*N*,*N*-bis(2-hydroxyethyl)propionamido aminoferrocene, Sal-NBAF (**23**).

Some of the recently reported electrochemical redox substrates for enzyme/protein biomarkers are summarized in Figure 5. Table 1 summarizes the analytical parameters of the electrochemical redox substrates reported for enzymatic biomarkers.

**Table 1:** Summary of Analytical Parameters of Electrochemical Redox Substrates to Determine Enzymatic Biomarkers^a^

biomarkers	substrate	reporter	trigger	linear range	lod	real sample	assay time	technique	pros	cons
β-gal^20^	**1**	hydroquinone	glycosidic unit	0.21–1.64 U	0.06 U	calf thymus	30 min	CV	reagent-less, selective, rapid assay	tedious probe synthesis step tedious
β-gal^11^	**2**	4-methoxyphenol	glycosidic unit	12–1200 ng	5 ng	*E. coli*	1 h	DPV	live cell analysis, no pretreatment	longer assay time
β-gal^21^	**3**	4-aminophenol	glycosidic unit	10^2^–10^7^ CFU	10^3^ CFU	water, apple juice, milk	3 h	DPV	high specificity toward bacteriophages	probe itself shows background signal
β-gal^15^	**4**	4-nitrophenol	glycosidic unit	10^2^–10^7^ CFU	1.5 μg	spinach, sprouts, water	4 h	DPV	both colorimetric and electrochemical signals	longer analysis time
β-gal^22^	**5**	aminoferrocene	glycosidic unit	0.1–10 U			1 h	DPV	reporter signal at interference-free region	not tested in real-time assay
ALDA^23^	**6**	aminoferrocene	aldol, retro aldol units	0.33–6.66 μM				CV, AMP	rapid and easy detection	not tested in real time assay
ALP^30^	**7**	aminoferrocene	phosphate ester unit	5–1000 U	0.4 U		30 min	DPV, AMP	water-soluble substrate commercial feasibility	higher pH working medium
ALP^31^	**8**	paracetamol	phosphate ester unit	10^–4^-1 U	0.02 U	mouse samples	15 min	AMP	PoC glucometer assay	requires 37 °C
LE^34^	**9**	hydroquinone	sulfonyl ester	9–690 μg	9 μg/mL	blood cells	15 min	CV	quantitative diagnosis of infection	multistep synthesis
NA^39^	**17**	glucose	sialic acid	10^2^–10^8^ PFU	10^2^ PFU	Influenza virus	2 h	AMP	commercial glucose strips integration	long assay time
NA^40^	**18**	4-aminophenol	sialic acid	15–300 ng	5.6 ng	blood, nasal swab, urine	30 min	CV	simple synthesis steps	background signal of reporter.
SHL^42^	**19**	aminoferrocene	salicylate, NADH	35–560 μM	5 μM	whole blood and serum	30 min	CV, DPV	no pretreatment	less product conversion
LAP^43^	**20**	aminoferrocene	leucine residue	200–1200 ng	2 ng	HepG2 cells	30 min	CV, DPV	low sample volume, live cell studies	requires sample pretreatments
LAP^44^	**21**	*N*-aminoferrocene	alanine residue	0.05–110 ng mL^–1^	23.18 pg mL^–1^	HepG2 cells, blood, urine	30 min	CV, DPV	simple synthesis, no pretreatments	antifouling issues
SE^45^	**22**	CAF	C8-aliphatic ester	1.03 × 10^5^–1.1 × 10^10^ CFU	39.27 × 10^3^ CFU/mL	milk, bacteria	7 h	CV, DPV	easy probe synthesis, selective	long assay time
SE^45^	**23**	NBAF	C8-aliphatic ester	0.09–0.13 U	1.92 × 10^–3^ U	bacteria	7 h	CV, DPV	easy probe synthesis, selective	long assay time

a Abbreviations: β-gal: β-glycosidases; ALDA: aldolase; ALP: alkaline phosphatase; LE: leukocyte esterase; NA: neuraminidase; SHL: salicylate hydrolase; LAP: leucine amino peptidase; SE: Salmonella esterase; CAF: carboxyl aminoferrocene; NBAF: *N*,*N*-bis(2-hydroxyethyl)propionamido aminoferrocene; NADH: nicotinamide adenine dinucleotide; LOD: limit of detection; CV: cyclic voltammetry; DPV: differential pulse voltammetry; AMP: amperometry; PFU: plaque-forming unit; CFU: colony-forming unit.

### 3.1 β-Galactosidase

**Figure 6:**
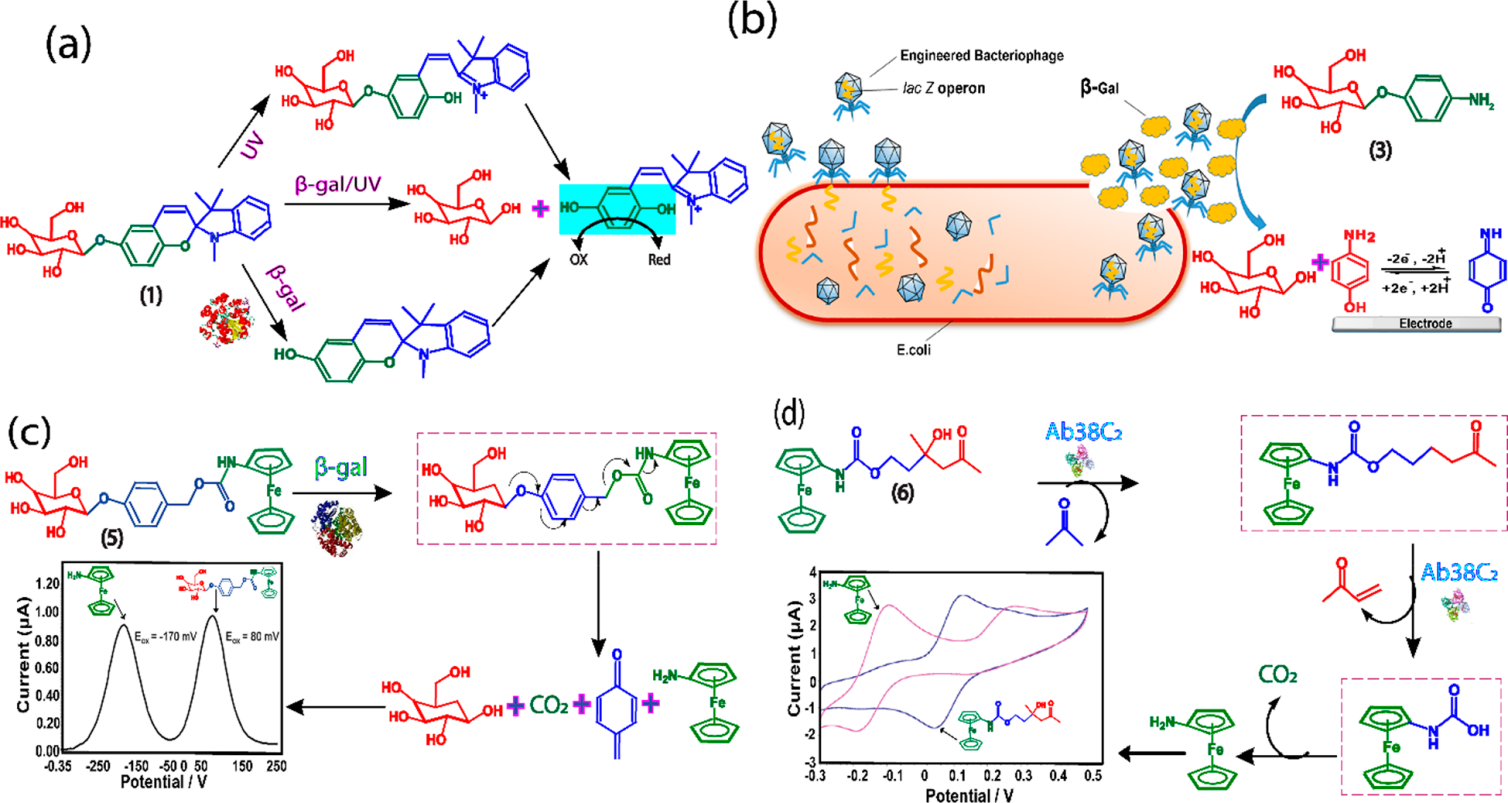
(a) Electrochemical design and working principle of SP-β-gal (**1**). Reprinted with permission from ref (20). Copyright 2014 Elsevier. (b) Electrochemical detection of *E. coli* from aqueous samples using a substrate PAPG **(3)**. Reprinted from ref (21). Copyright 2017 American Chemiocal Society. (c) Working principle of substrate FCPG (**5**) to sense β-gal and DPV of the substrate and reporter. Reprinted with permission from ref (22). Copyright 2017 Royal Society of Chemistry. (d) Electrochemical working principle of substrate FAC (**6**), its hydrolysis mechanism with aldolase, and cyclic voltammograms (CVs) of FAC and reporter. Reprinted from ref (23). Copyright 2006 American Chemical Society.

β-Glycosidases (β-Gal) are a primary reporter gene and a vital hydrolase enzyme that promotes the glycolytic binding of carbohydrates with water. It serves as an important regulator enzyme and plays an important role in genetics, molecular biology, and other biological processes. β-Gal is widely used as a marker for enzyme-linked immunosorbent assays (ELISA) and fecal coliform determination via observing their transcriptional regulation and genetic expression/inhibition. The subgroup of glycosidases and β-gal are involved in many physicochemical processes and cleave the glycosidic bond from the β-galactose into a sugar unit.^19^ A handful of activity-based electrochemical substrates have been developed for β-gal using β-Gal cleavage of the glycosidic linkage because of the luxury of using glycosidic linkage as a trigger as well as a linker. To avoid nonspecific interactions of targeting mediators and signal distortion/overlap, Tao et al. developed an activity-based β-gal targeting spiropyran-based electrochemical substrate, SP-β-gal (**1**), consisting of a merocyanine ring attached to the galactose moiety at the 6-position via a glycosidic bond (β-gal hydrolytic moiety).^20^ Under optical stimulation, the closed form of the merocyanine ring opens to form a phenolic oxygen anion, which is converted to the electroactive substance hydroquinone (reporter) by enzymatic hydrolysis of β-Gal (Figure 6a). The ratiometric redox signal was found to increase at 0.33 V (Ag quasi-reference electrode), indicating a well-defined one-electron transfer reaction of hydroquinone–quinone. In addition, the authors self-assembled SP-β-gal on a single-walled carbon nanotube (SWCNTs)/glassy carbon electrode (GCE), and this approach amplified the detection signal. The method was successfully demonstrated in cell culture media. Although the probe is specific to β-gal, the method requires external UV-irradiation to activate the β-gal hydrolysis process and to eject the redox reporter. Our group developed a novel off-on substrate, 4-methoxyphenyl-β-galactopyranoside (4-MPGal) (**2**) using the self-immolative linker approach, which is spontaneous upon incubating the probe and β-gal not requiring any external initiator like UV-irradiation.^11^ The 4-MPGal substrate typically contained its unique trigger group and masked electrochemical reporter connected via self-immolative linker.^11^ The working principle is based on the expulsion of the masked reporter from the latent substrate, provoked the elimination of substrate-analyte communication, and unmasked the redox active site is highly specific toward β-gal enzyme. The 4-MPGal substrate consists of a 4-methoxyphenol reporter and galactose unit linked by a glycosidic bond that does not emit a background signal. When it interacts with the β-gal, a new peak emerges at +0.50 V (Ag/AgCl), corresponding to the electrochemical signal of 4-methoxyphenol. This probe can be used for *real-time* monitoring of the β-Gal expressions in *E. coli*.

In another work, Wang et al. reported an electrochemical substrate, 4-aminophenyl-β-galactopyranoside, PAPG (**3**) for β-Gal, in which galactose units bind to glycosidic bonds with 4-AP electrochemical reporter.^21^ This substrate provides a relatively low background signal that allows chemical modification to form free 4-AP after treatment with β-Gal. The 4-AP product exhibited distinct redox peaks at a slightly low overpotential region (+ 0.20 V vs Ag/AgCl), which can be attributed to the reversible one-electron/one-proton transfer reaction of 4-AP on the electrode surface. Using differential pulse voltammetry (DPV) as a signal read-out, the endogenous and phage-induced β-gal was detected using the electrochemical method with PAPG as a substrate (Figure 6b). Adkins et al. demonstrated various substrates containing different reporters for detecting β-gal and β-glucuronidase (β-glucr) and used this method to detect *E. coli* and *enterococcus species*, both indicators of fecal contamination. *p*-nitrophenyl-β-galactopyranoside, PNPG (**4**), was found to perform well for β-gal. It also offers colorimetric detection capabilities that are more useful in cultured bacterial cells and real pathogenic and nonpathogenic samples that can be monitored by a paper-based smartphone system (Figure 6c).^15^ In addition, the authors tested various other substrates, including *p*-nitrophenyl-β-d-glucopyranoside (PNP-Gluco), *p*-nitrophenyl-β-d-glucuronide (PNP-glucr), *o*-nitrophenyl-β-d-galactopyranoside (ONPG), *p*-aminophenyl-β-d-galactopyranoside (PAPG), and *o*-nitrophenyl-β-d-glucopyranoside (ONP-Gluco). The reporters are either *p*-nitrophenol, *o*-nitrophenol, or *p*-aminophenol. The authors studied the electrochemical performance of each substrate and reporter and optimized the right probe and reporter of each enzyme based on their analytical performances.

Ferrocene-based electrochemical sensing continues to be of interest to analytical researchers as it allows for inexpensive, simple synthetic steps and a convenient way to monitor β-gal enzyme activity. Accordingly, Sam et al. established a substrate (**5**), ferrocenylcarbamoylphenyl-β-D-galactosidase (FCPG) to measure β-gal activity.^22^ The FCPG consists of aminoferrocene electrochemical reporter and benzyl alcohol linked galactose as the β-gal recognition unit. The substrate undergoes an unstable anionic phenolate intermediate by β-Gal-induced hydrolytic cleavage and subsequent 1,6-quinone methide elimination would release the free aminoferrocene. The electron-poor FCPG ferrocene carbamate would have a more positive oxidation potential (+ 0.08 V, Ag/AgCl) than the electron-rich aminoferrocene (−0.17 V, Ag/AgCl), making them electrochemically distinguishable, allowing ratiometric electrochemical analysis of β-gal activity.

Overall, spiropyran based probe, SP-β-gal (**1**), 4-methoxyphenyl-β-galactopyranoside, 4-MPGal (**2**), 4-aminophenyl-β-galactopyranoside, PAPG (**3**), *p*-nitrophenyl-β-galactopyranoside, PNPG (**4**), and ferrocenylcarbamoylphenyl-β-d-galactosidase (FCPG) (**5**). All of them are stable at room temperature, have a longer shelf life, and are selective to β-gal. PAPG and PNPG are commercially available, and others are easy to synthesize with a single or few steps. Interestingly, *p*-aminophenol and *p*-nitrophenol reporters are electrochemically active as well as colorimetric active, which can provide an option to develop a sensing platform with multiple mediums of signal read-out. Adkins et al. demonstrated successful integration of the electrochemical substrates with paper-based wells and transparency-film electrochemical cells, which indicates the electrochemical substrates hold potential for large-scale industrial and point-of-care applications. The FCPG substrate provides a ratiometric sensor, in which the electrochemical signals of both reporter and substrate can be used for detecting β-gal, which is good for increasing accuracy. In addition, the detection potentials of aminoferrocene and FCPG are observed at close to 0 V, meaning that biological interferences can be eliminated. However, the incubation time is longer, ranging from 30 min to 4 h which must be improved. Also, not all the probes are soluble in aqueous solutions, requiring the use of organic solvents that are not sometimes compatible with enzymes.

### 3.2 Aldolase

Aldolase enzymes, especially aldolase A, are overexpressed in tumor cells, and are clinically important biomarker for a variety of cancer, including colorectal cancer, lung squamous cell carcinoma, renal cancer, and hepatocellular carcinoma.^24^ Aldolase blood test has been widely used for the diagnosis of muscle weakness including myositis–a type of autoimmune disease. Amit et al. developed a sensitive and selective electrochemical redox substrate (**6**), ferrocene-aldol carbamate (FAC), for measuring aldose activity, using *aldose antibody 38C2* as a representative model.^23^ Because both the signals of substrate and reporter are linearly responding with Aldolase, a ratiometric sensor is developed. The substrate (**6**) consists of a carbamate moiety covalently linked to a ferrocenamine that generates free reporter aminoferrocene by hydrolysis of the aldose enzyme through retro-aldol-retro-Michael activity (Figure 6d). The kinetic constants *K*_m_ and *k*_cat_ were calculated to be 375 ± 19 μM and 0.24 ± 0.012 min^–1^, respectively, which agree with the other Michaelis–Menten constants measured for similar substrates of antibody 38C23, indicating the potential of this substrate for assaying aldolase. In addition, the substrate showed biocompatibility and sensitivity and was able to selectively detect aldose. The detection potential region was several millivolts away from the typical biological interference-causing region, this potential shift is caused by the iron core redox reaction of (**6**) encompassed by the low electron density and the AF surrounds the high electron density which renders a selective detection.^23^ But, the synthesis procedure of this substrate involves multiple steps, limiting its practical applications. In addition, the stability of this substrate is not provided.

### 3.3 Alkaline Phosphatase

**Figure 7:**
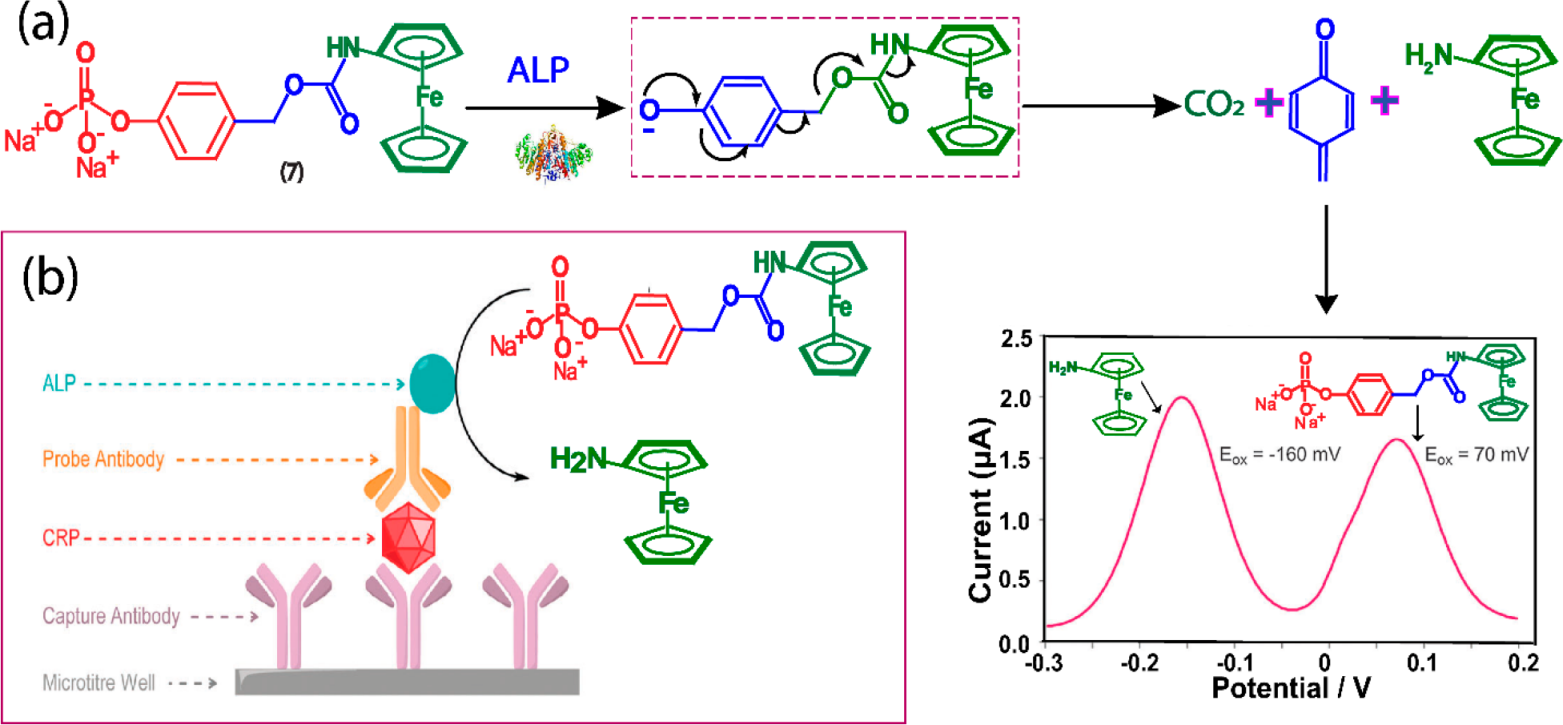
(a) Electrochemical design and working principle of ferrocenylphenyl phosphate (**7**). Sensing of ALP using substrate (**7**) and hydrolytic redox release of aminoferrocene recorded by DPV.^30^ (b) Pictographic model of ELISA-based ALP-labeled CRP immunoassay using the substrate (**7**). Reprinted with permission from ref (30). Copyright 2015 Royal Society of Chemistry.

Alkaline phosphatase (ALP) is an extensively analyzed enzyme for the diagnosis of several diseases, including liver dysfunction, kidney injuries, and bone diseases. In addition, ALP is one of the most frequently used enzyme labels for ELISA because of its reliability and compatibility with antibodies. Recently, research in this area has made significant progress, especially in the development of electrochemical substrates for the enzyme ALP. Phenyl phosphate ester can be used as an electrochemical trigger for ALP and therefore has a good specificity and ability to dephosphorylate or transphosphorylate designed substrate.^25^ The most commonly used redox substrate for ALP is l-ascorbic acid-2-phosphate (AA2P), which converts ascorbic acid redox reporter via electrochemical reaction.^26,27^ However, the electroactive redox reporter generates its optimal current only in an acidic medium, which does affect the sensitivity. A few ferrocene-based studies have been reported for the ALP activity assay, including N-ferrocenoyl-4-aminophenyl phosphate by Bannister et al.^28^ and 6-(*N*-ferrocenoylamino)-2,4-dimethylphenyl phosphate by La Gal La Salle et al.^29^ Nevertheless, both the substrate and the ALP-catalase produced the same electron accepting products, although no detectable signal was generated by exchanging the phosphate moiety into an alcohol moiety, resulting in poor sensitivity due to the sluggish electron transfer. To overcome this issue, Goggins et al. developed an electrochemical substrate, ferrocenylphenyl phosphate (**7**) using the activity-based self-immolative linker strategy for ALP activity determination.^30^ This described substrate consists of a phosphate group and a ferrocene-amine reporter connected via a self-immolative linker. The substrate exhibits excellent hydrolytic ability toward ALP, with dephosphorylation leading to an unstable anionic phenolate intermediate and consequently releasing aminoferrocene reporter via 1,6-quinone methide elimination along with byproducts. Here, the electrochemical signal of (**7**) was observed at a positive potential of +70 mV and the signal of the reporter was observed at a lower potential of −160 mV. As a result, the signal of the reporter can be electrochemically distinguishable from its substrate by 230 mV and the authors able to monitor the ALP activity ratiometrically (Figure 7a). The developed substrate was further applied for the ratiometric electrochemical detection of C-reactive protein (CRP) through ALP labeled ELISA because of its high sensitivity (Figure 7b).

More recently, Sheng et al. developed another redox activity substrate (**8**), based on a paracetamol-bearing substrate, 4-acetamidophenyl phosphate that reacts with ALP to cleave acetaminophen, which further oxidized directly on the detector’s electrode surface and is sensed by the glucometer.^31^ Interestingly, the author coupled the electrochemical substrate methodology with a commercial glucometer and demonstrated a point-of-care (PoC) analysis to detect intestinal alkaline phosphatase which is a potential biomarker to monitor. The assay demonstrated good selectivity and sensitivity with a detection limit of 0.11 U within 15 min of hydrolysis time under physiological conditions.

### 3.4 Leukocyte Esterase

A common urinary infection is diagnosed by the detection of the leukocyte esterase (LE) enzyme, which is mainly used as a urine test marker to monitor active white blood cells and other abnormalities associated with the disease. LE has a wide range of different substrate specifications, protein structures, and biological functions.^32^ The chromogenic LE dipsticks are the most widely used diagnostic tool for quick evaluation of infections. However, they are generally qualitative and suffer from limited resolution and low sensitivity, limiting their reliability in making decisions.^33^ Gorski’s research group demonstrated that electrochemical redox reporters can overcome these issues and hold the potential to offer a reliable assay for LE. For example, Gorkski et al. reported a substrate (**9**), 4-((tosyl-l-alanyl)oxy)phenyl tosyl-l-alaninate (TAPTA) for a quantitative LE assay as a high-resolution alternative to existing optical LE assays. TAPTA contains a sulfonyl ester triggering unit and a hydroquinone reporter connected via an l-alanine linker that elicits a specific response to the LE enzyme.^34^ In the presence of LE, the ester group induces substrate **9** to release hydroquinone, which then produces its electrochemical signal. As a LE substrate, TAPTA showed Michaelis constant *K*_m_ and *I*_max_ of 0.24 mM and 0.13 mA/cm, respectively. The assay was practically demonstrated in saliva samples via internally calibrated electrochemical continuous enzyme assay. However, substrate **9** requires the use of DMSO for its solubility. The same group developed three additional derivatives **9**, namely, **10**–**12**, to improve water solubility and to increase electrocatalytic activity.^33,35^ The detailed studies of these probes with LE indicate they are useful for rapid, high-resolution, and sensitive measurements of LE activity.

Alongside, Bekhit et al. designed and synthesized four glucosyl esters **13**–**16** for LE.^36^ The interesting part of these probes is the use of glucose as the signaling molecule. As a result, the substrate can be easily coupled with commercial glucose meters, making this method highly reliable and universally acceptable. The glucosyl esters-based substrates **13**–**16** release glucose upon interaction with LE in direct proportion to the activity of LE. The liberated glucose can be detected with any glucose sensors. In this work the author demonstrated two types of glucose sensors, one is with a commercial glucose test strip, and another is with a nitrogen-doped carbon nanotube electrode-based glucose biosensor. When used with glucose strips, these electrochemical substrates can detect clinically relevant levels of LE up to 26 nM (800 μg L^–1^) in microliter-sized samples of bodily fluids. These electrochemical substrates, when combined with glucose strips, can detect clinically significant levels of LE up to 26 nM (800 g L^–1^) with low sample volume (μL range). The same group demonstrated the use of a commercial glucose meter. Bekhit et al. proposed methyl pyruvate as an electrochemical substrate for LE.^37^ The reaction of methyl pyruvate and LE was coupled with alcohol oxidase to release hydrogen peroxide, which was detected using a nitrogen-doped carbon nanotube electrode at −0.20 V (vs Ag/AgCl), yielding a current signal proportional to the concentration of LE in the sample.^37^

Overall, hydroquinone and glucose-based electrochemical substrates have been reported for LE so far. Though some substrates require organic solvents, the following research indicates synthesizing aqueous soluble LE substrates are easy and not complicated. Incubation times are significantly reduced, somewhere between 5 and 30 min, which is highly attractive compared to 4 h of ELISA tests.^34,37^ The most interesting development is the integration of the LE substrates with commercial glucose strips which increases reliability, accuracy, and sensitivity, indicating that LE coupled glucose stirps are an interesting approach to overcome the limitations currently faced by chromogenic LE dipsticks. However, the LE coupled commercial glucose strips reported are yet to be validated with patient samples. Besides, most of the substrates are tested with traditional electrodes such as glassy carbon electrodes, which are not suitable for PoC applications or not comparable with existing LE dipsticks.

### 3.5 Neuraminidase

**Figure 8:**
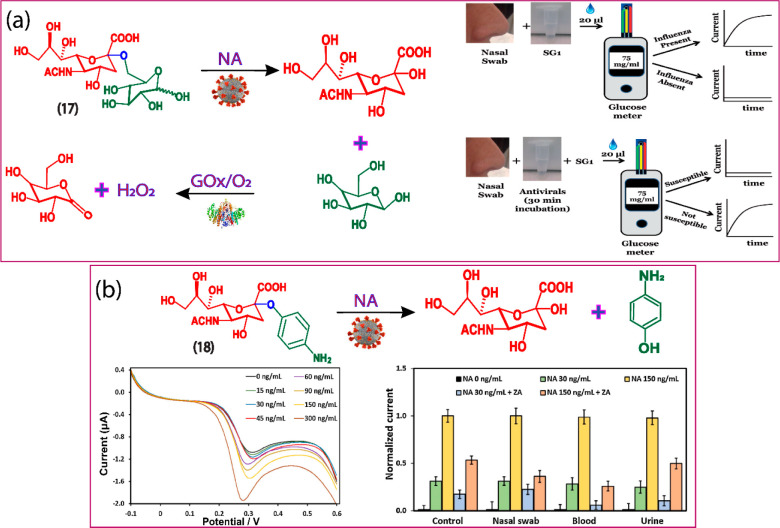
(a) Hydrolysis and sensing of neuraminidase (NA) using substrate **17**. Reprinted with permission from ref (39). Copyright 2015 Wiley. (b) Electrochemical working mechanism and CV monitoring of substrate **18** in the presence of neuraminidase. Reprinted with permission from ref (40). Copyright 2017 Elsevier.

Neuraminidase is found on the surface of influenza viruses and pathogenic bacteria and contributes to the spread of the virus from the infected host cell. Neuraminidase is considered a class of enzymes that degrade the terminal sialic acid group of glycoproteins, glycolipids, and oligosaccharides.^38^ The biochemical activity of neuraminidase does not appear to be satisfactory in monitoring its biological activity during its viral life cycle. Physiological variations in neuraminidase function may therefore be important from a diagnostic point of view. Zhang et al. developed an electrochemical substrate, sialic acid derivate (SG1), **17**, using glucose as the reporter.^39^ In the structure of **17**, the sialic acid moiety is directly bound to glucose at the 6-position by an ether bond, recognizing the hydrolytic site of neuraminidase with excellent sensitivity and selectivity, and cleaving the ether bond to release free glucose, which is measured amperometrically by the glucose test strips (Figure 8a). About 19 different influenza strains were successfully detected using **17** coupled with commercial glucose strips. The detection limit (LOD) and linear ranges are 10^2^ and 10^2^–10^8^ plaque-forming units (pfu), respectively. The results of **17** are validated with rRT–PCR and plaque assays. The sample volume is 20 μL, and the substrate is soluble in aqueous solutions, which are ideal for clinical analysis. However, the incubation time is 1 h, which requires further research. The probe is aqueous soluble. By introducing larger groups at the 4-position of the sialic acid, substrate **17** can be made highly specific for viral neuraminidase distinguished from bacteria/human neuraminidase. Because bacteria/human neuraminidase have a smaller binding pocket and cannot accommodate larger groups at the 4-position of sialic acid. This means that there are a lot of opportunities to functionalize the substrates based on the demand from the diagnostics side. On the contrary, such functionalization options are limited to antibodies and proteins.

In continuation, our group developed a new class of activity-based redox substrate **18**, *N*-acetyl-2-*O*-(4-aminophenyl)-neuraminic acid (AP-Neu5Ac) for highly selective detection of neuraminidase, which is successfully tested in spiked blood, urine, and nasal swab samples. The substrate **18** is equipped with a unique sialic acid recognition unit connected to redox reporter 4-aminophenol. In the presence of neuraminidase, substrate **18** cleaves the sialic acid bond and releases the reporter. The electrochemical DPV signal of the reporter can be monitored, which revealed highly intense signals at +0.30 V (vs Ag/AgCl), which is highly specific to neuraminidase (Figure 8b).^40^ The assay was highly sensitive with an LOD of 5.6 ng mL^–1^, which is comparable to the ELISA and luminescence-based assays. The substrate synthesis is a rapid, simple one-step hydrogenation reaction.

Most of the current clinical sensors for screening viruses depend on qualitative analysis. The quantitative analysis of viruses is usually dependent on ELISA, which requires at least 4 h, extensive washing steps, and requires a highly skilled technician. In contrary, electrochemical substrates such as **17** and **18** provide a rapid assay (30 min-1 h) and simple incubation steps, the substrates are highly stable at room temperature, and user-friendly. Unlike substrate **17**, our substrate **18** is not compatible with commercial glucose meters because of the use of *p*-aminophenol reporter. However, substrate **18** provide a much-improved detection limit compared to **17**. In addition, the incubation time of **18** is 30 min which is faster than **17** (requires 1 h incubation time). Further research can be directed toward immobilizing these neuraminidase electrochemical substrates on solid supports such as strips, which are more familiar to the common people and easy to be adopted by the people and clinicians.

### 3.6 Salicylate Hydrolase

Salicylate hydrolase (SHL) or salicylate-1-monooxygenase (SMO) is an oxidoreductase enzyme that binds to salicylate and NADH to form a reduced enzyme–substrate complex, which then reacts further with O_2_ to form catechol, H_2_O, and CO_2_.^41^ The details of the reaction are as follows: Salicylate + NADH + O_2_ + 2 H^+^ ↔ Catechol + NAD + + H_2_O + CO_2_. This reaction is particularly active in the presence of a pair of donors, O_2_ as an oxidant, and the incorporation or reduction of oxygen. SHL has been explored as an immobilized sensing unit on electrodes to develop general-type biosensors that produce electrochemical properties. Consumption of NADH/oxygen or formation of catechol/CO_2_ can be monitored and is proportional to salicylate concentration. Pathogens have developed ways to manipulate SHL-mediated defense signals from the cells of microorganisms. Monitoring the nature of SHL interactions is essential for determining the mechanisms of its activation. To study SHL activity, our group developed a new electrochemical redox substrate **19**, salicylic acid amino ferrocene (SAF).^42^ The chemical composition of SAF consists of a salicylate moiety with a carbamate linker bound to a reporter of aminoferrocene. SHL promotes decarboxylate hydroxylation in the presence of NADH under aerobic conditions on the substrate SAF, and the quinone methide-type regeneration reaction releases the reporter that can transfer its redox signals from higher (+0.22 V vs Ag/AgCl) to the lower potential at (−0.10 V vs Ag/AgCl). As a result, a ratiometric sensor can be developed by monitoring the disappearance of SAF’s signal and the emergence of reporter aminoferrocene’s signal. Substrate **19** is not only useful to assay SHL but also can be extended for the determination of salicylic acid and β-hydroxybutyrate. The electrochemical assay developed for SHL avoids cumbersome steps such as, enzyme immobilization and pretreatments.

### 3.7 Leucine Amino Peptidase

**Figure 9:**
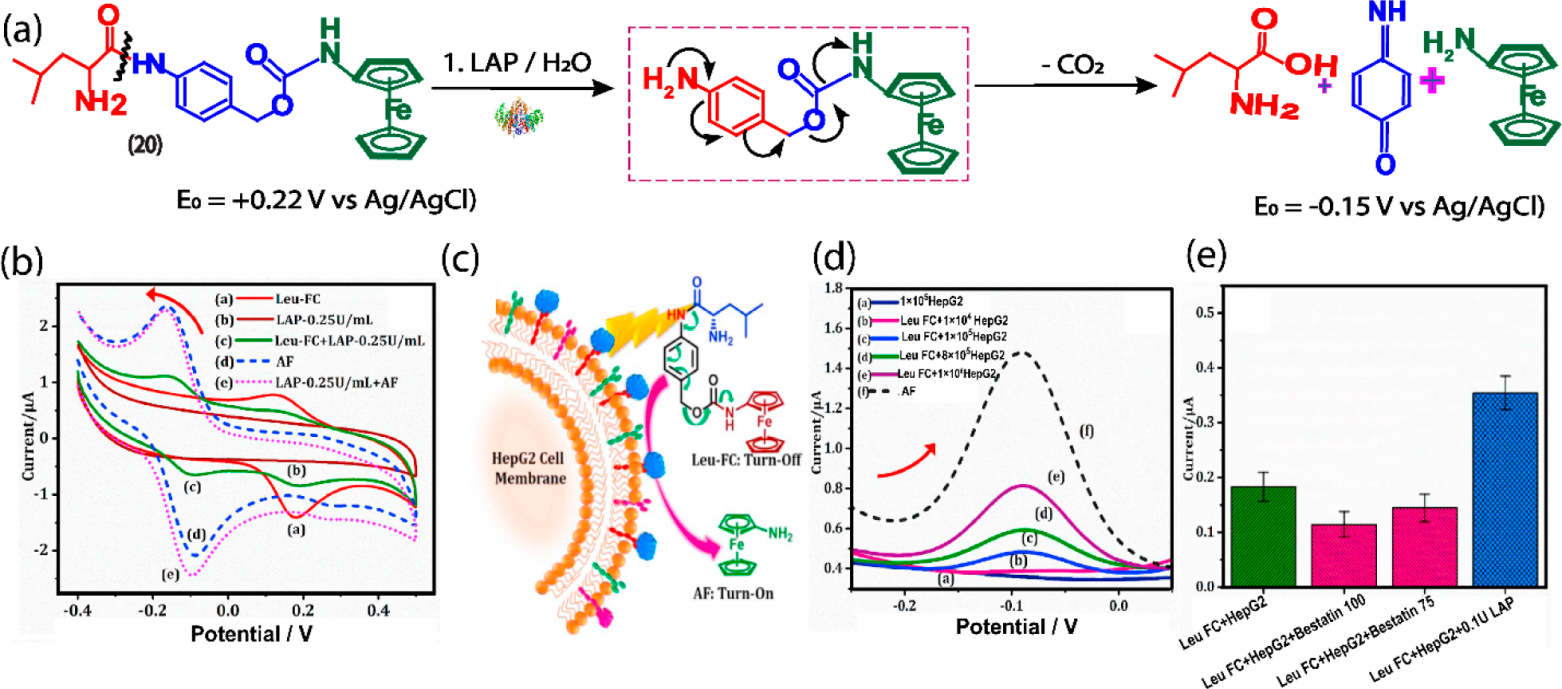
(a) Electrochemical working mechanism substrate **20**, Leu-FC in the presence of LAP.^43^ (b) CV monitoring of Leu-FC substrate in the presence of LAP, (c) LAP catalysis under cell medium using **20**. (d,e) DPV response of cultured samples assay using **20**. Reprinted with permission from ref (43). Copyright 2020 Elsevier.

Leucine amino peptidase (LAP), also known as amino peptidase N (APN), is a zinc-dependent metalloprotease that belongs to the M1 family of ecto-enzymes. LAP contains their extracellular sites, including PepA, Lap, and Lap-A, which preferentially cleave neutral amino acids from the amino terminus of oligopeptides, especially leucine residues, and their bioactive substrates to release alanine. It is abundant in the kidneys and central nervous system and is used as a potential biomarker for the detection of liver malignancy and tumor cells. In this sense, our research group recently developed a novel electrochemical substrate **20**, leucine-benzyl ferrocene carbamate (Leu-FC) for the specific measurement of LAP activity in living systems.^43^ The substrate **20** consists of aminoferrocene as a redox reporter and l-leucine as a LAP recognition group connected via a carbamate linker (Figure 9). The substrate itself exhibits a signal in the positive potential region (+0.22 V vs Ag/Agcl) in the absence of LAP while exhibiting impressive selectivity and sensitivity to LAP by enzymatically cleaving the amide bond, thereby releasing a free amino group that leads to the degradation of the 1,6-iminoquinone cascade, which emits a linear electrochemical current aminoferrocene at -0.15 V (vs Ag/AgCl). The substrate was found successful in real-time active profiling of cellular LAP activity in HepG2 cells and the effect of LAP inhibitor. In addition, the substrate can effectively monitor cisplatin-induced overexpression of LAP activity in HepG2 cells in the presence and absence of bestatin. Unlike the traditional antibody-based immunoassays, substrate **20** based assay can monitor the in situ activity of LAP in live cells. We redesigned the substrate to develop another substrate **21**, Alanine-benzyl alkylated ferrocene carbamate (Ala-AFC) to sense LAP activity in an improved manner in the biosystem.^44^ The substrate **21** consists of an l-alanine recognition unit and an alkylated-ferrocenylamine redox reporter connected via a carbamate linker that undergoes selective hydrolysis at the l-alanine position, similar to l-leucine, to remove the amino-terminal unit, resulting in 1,6-iminoquinone self-immolative linker disconnection and consequently redox variation was observed in the negative potential region (−0.09 V). Substrate **21** showed excellent sensitivity for LAP detection, with a detection limit of 23.18 pg mL^–1^ and a working range of 0.05–110 ng mL^–1^, which are clinically useful levels.

Both substrates **20** and **21** showed promise for the ratiometric monitoring of active LAP in real-time. They are useful for specifically profiling cellular surface-expressed LAP, which is needed to understand and track the related physiological, and pathological functions. Because both substrate and reporters are electrochemically active, ratiometric monitoring at two potential windows is attained which is highly recommended to increase the accuracy of the sensor readings. Substrate **20** has additional advantages over **20** including high sensitivity, improved aqueous solubility, and good biocompatibility in live cells. However, both **20** and **21** require the use of organic solvent DMSO to prepare stock solutions. When high concentrations of either **20** or **21** are incubated with aqueous solutions of LAP, the formation of particles is observed causing aggregations. Only optimized threshold levels of substrate can provide reliable sensing results, meaning the structure of the substrates requires further redesign by introducing different functional groups to make them aqueous soluble. Another possible solution could be immobilizing the substrates directly on the electrode surface.

### 3.8 Salmonella Esterase

**Figure 10:**
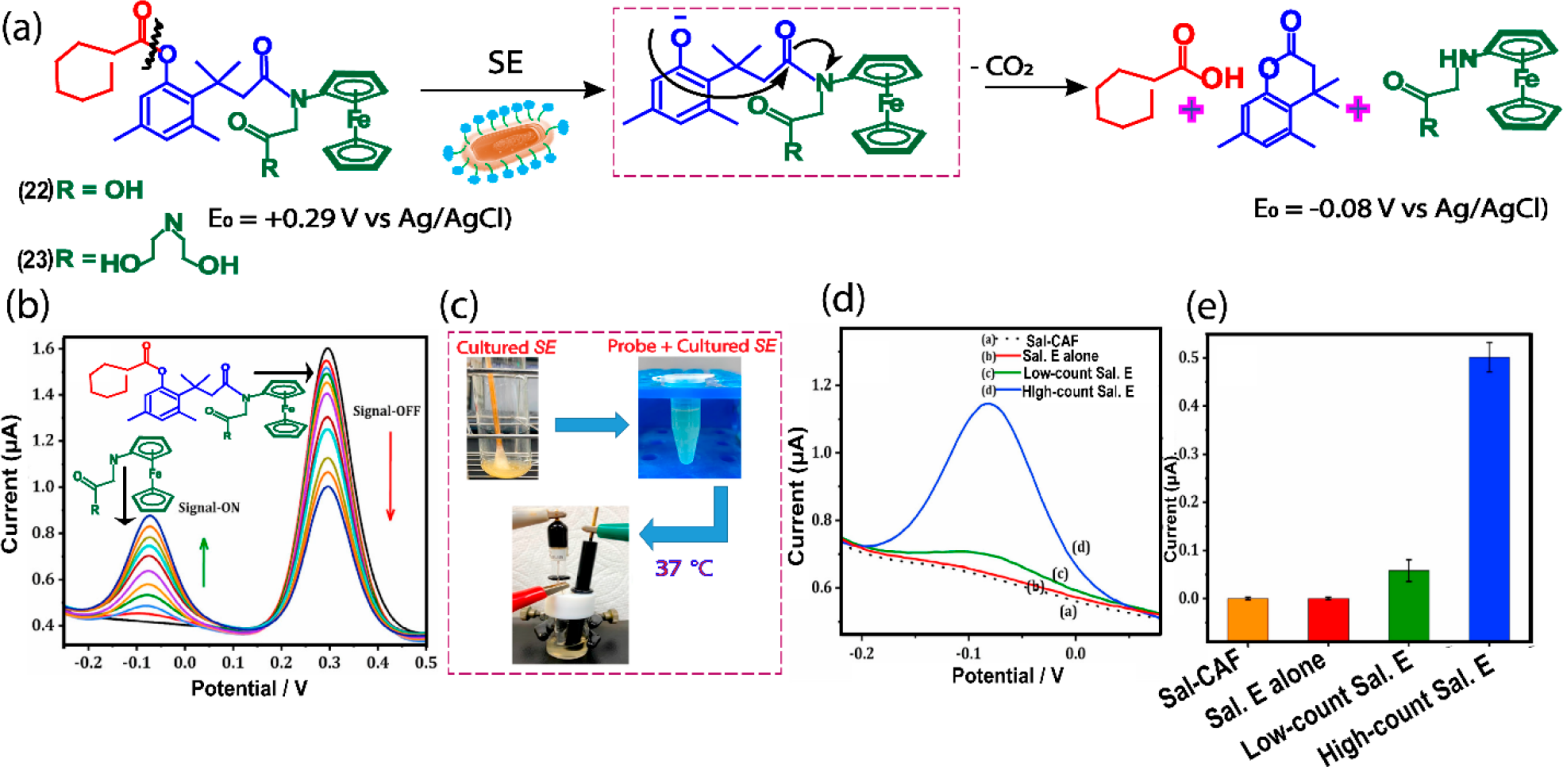
Electrochemical design and working mechanism **22** (Sal-CAF) and **23** (Sal-NBAF) substrates and their redox responses in the presence of *Salmonella esterase*.^45^ (b) CV observation of **22** substrate in the presence of *Salmonella esterase*, and (c–e) *Salmonella esterase* monitoring in live cells using **22**. Reprinted with permission from ref (45). Copyright 2022 Elsevier.

*Salmonella esterases* are a class of bacterial enzymes that are important human pathogens responsible for food contamination and cause zoonotic diseases. It catalyzes the hydrolysis of various endogenous and exogenous chemical substrates containing site-specific units of fatty acid naphthyl esters and C6 to C16 fatty acid phenyl esters, allowing selective biomarker detection of Salmonella. Recently we reported an electrochemical substrates **22** (carboxyl aminoferrocene, Sal-CAF) and **23** (*N*,*N*-bis(2-hydroxyethyl)propionamido aminoferrocene, Sal-NBAF), for the detection of Salmonella esterase activities directly in live virulent Salmonella by microbial culture samples.^45^ The difference between 22 and 23 is the types of reporters: **22** contains carboxyl aminoferrocene (CAF) as the reporter, while **23** contains *N*,*N*-bis(2-hydroxyethyl)propionamido aminoferrocene (NBAF) as the reporter. The purpose of installing different derivatives is to increase the electron richness around the substrate and to increase the distance between electrochemical signals of the substrate and reporter which makes the ratiometric sensor more accurate. The probe consists of aminoferrocene as the electrochemical reporter unit and a trimethyl lock-based self-immolative linker connected to a C8 ester (octyl ester), which triggers each probe, as the Salmonella recognition unit (Figure 10a). The probe undergoes *Salmonella esterases*-induced hydrolysis, which triggers rapid intramolecular cyclization to release the reporter. The electrochemical potential of the reporter is negatively shifted compared to that of reporters **22** and **23**, allowing a ratiometric electrochemical current response at two potential windows, - 0.08 V and +0.29 V (vs Ag/AgCl) (Figure 10b–e). The potential window of the substrate provides a signal-off signal, while the potential region of the reporters provides a signal-on signal, thus ratiometric ON-OFF signals can be attained. The substrate **22** offered additional advantages such as improved hydrophilicity and sensitivity than **23** due to a highly polar carboxylic acid functional group at the amino-N position of **22**.

## 4 Self-Immolative Redox Switches for Non-enzymatic Biomarkers

**Figure 11:**
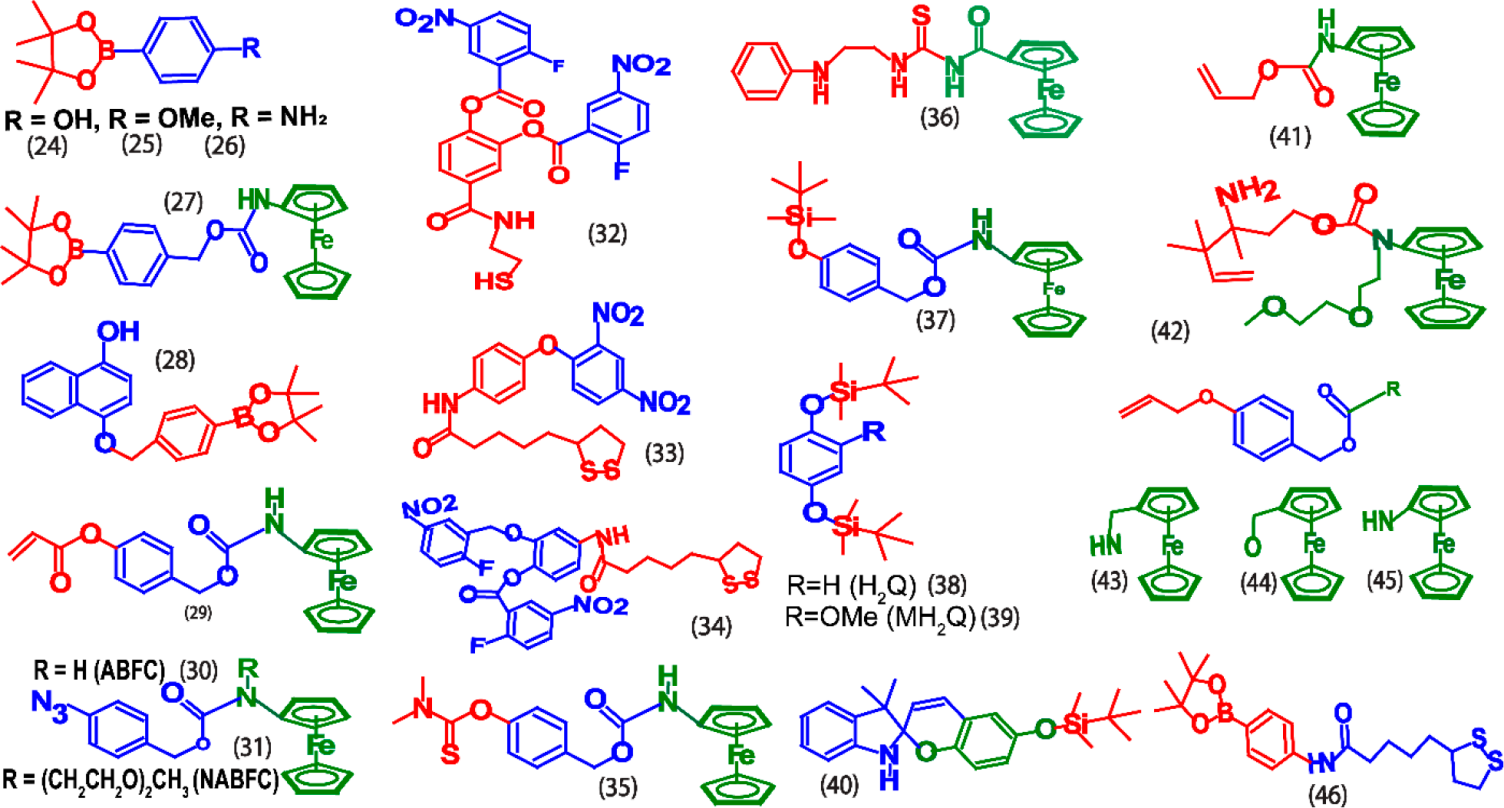
Chemical structures of electrochemical substrates (**24**–**46**) for nonenzymatic biomarkers.

This section provides an overview of self-immolative electrochemical redox substrates developed for nonenzymatic biomarkers. This set of biomarkers includes both electrochemically active and inactive molecules. Especially, this approach is more useful for the analytes that are electrochemically inactive molecules such as glucose, creatinine, formaldehyde, and phosphine. Several electrochemically active molecules show their electrochemical signals at a potential window that is more susceptible to biological interferences. For example, hydrogen peroxide, salicylic acid, etc., shows signal beyond +0.50 V (vs Ag/AgCl), which are not suitable for practical applications. With the use of the electrochemical substrate method, the detection potential can be shifted toward a potential window where minimized interference is possible. For instance, direct electrochemical sensing of H_2_O_2_ generally occurs at around +0.60 V (vs Ag/AgCl), it can be detected at −0.20 V (Vs. Ag/AgCl) with the help of electrochemical redox substrates.^14^ Metal ions are commonly detected by potentiometric methods; however, the sensitivity and detection limit of potentiometric methods are poor compared to that of voltammetric methods. Interestingly, voltammetric sensing methods can be established for detecting metal ions such as fluoride with the help of electrochemical redox substrates. The structures of the electrochemical substrates developed for various types of nonenzymatic biomarkers and analytes are sketched in Figure 11 and their analytical parameters are compared in Table 2.

**Table 2:** Analytical Parameters of Electrochemical Redox Substrates Reported for Non-enzymatic Biomarkers

biomarkers	substrate	reporter	trigger groups	linear range	lod	real sample	assay time	technique	pros	cons
H_2_O_2_^13^	**24**	hydroquinone	boronic acid	1.0–1000 μM	0.31 μM		1 min	CV	simple synthesis	no detailed studies
H_2_O_2_^48^	**25**	4-methoxyphenol	boronic acid pinacol ester	0.01–100 μM	4.1 nM	Caco-2, MCF-7 cells	30 min	CV, DPV	highly sensitive, selective	bulky electrode
H_2_O_2_^49^	**26**	*p*-aminophenol	boronic acid	0.001–1 mM	0.8 μM	honey samples	60 s	CV	simple synthesis	selectivity not validated
H_2_O_2_^14^	**27**	aminoferrocene	boronic acid ester	0–1000 μM			20 min	DPV	highly sensitive and selective	high pH use, requires organic solvent
H_2_O_2_^50^	**28**	naphthoquinone	boronic ester		0.5 μM		<1 h	CV	exponential amplification	complex reactions
H_2_O_2_^51^	**46**	*p*-aminophenol	boronic ester	0.5–400 μM	0.02 μM	whole blood	40 s	DPV	fast analysis time	complex electrode modification steps
glucose^14^	**27**	aminoferrocene	boronic acid ester	0–10 mM			20 min	CV	excellent sensitivity	high pH medium
salicylate^42^	**19**	aminoferrocene	salicylate moiety	35–560 μM	5.0 μM	whole blood	10 min	CV, DPV	highly sensitive, selective	cofactor dependence
cysteine^52^	**29**	aminoferrocene	acrylate moiety	10–200 pM	1.0 pM	W3110, whole blood	10 min	CV, DPV	simple, highly sensitive	multistep synthesis
H_2_S^10^	**30**, **31**	aminoferrocene	carbamate linkage	0.4–100 μM	76 nM	*E. coli*	30 min	CV and DPV	practicality in live cells	multistep synthesis of substrate
H_2_S*_n_*^53^	**32**	pyrocatechol	2-fluoro-5-nitrobenzoyl	0.2–50 μM	40 ± 3 nM	live mouse brain	50 s	DPV	substrate attached to electrodes	multistep substrate synthesis
H_2_S^53^	**33**	aminophenol	dinitrophenyl ether	0.2–40 μM	47 ± 4 nM	live mouse brain	50 s	DPV	substrate attached to electrodes	multistep substrate synthesis
H_2_S_n_^54^	**34**	pyrocatechol	2-fluoro-5-nitrobenzoyl	0.25–20 μM	50 nM	mouse brain	4 min	DPV	high selectivity	requires electrode pretreatment
HClO^55^	**35**	aminoferrocene	dimethyl thiocarbamate	0.35- 12 μM	75 nM	RAW 264.7	15 min	CV, DPV	easy synthesis steps	solubility issues
Hg^58^	**36**	ferrocene	thiourea	0–2 equiv of Hg^2+^	1.0 equiv of Hg^2+^	—	—	CV, DPV	highly selective	detailed studies not done
fluoride^56^	**37**	aminoferrocene	silyl moieties	1–100 μM	5.1 × 10^–7^	bovine serum, toothpaste	1 h	CV, DPV	simple; Highly sensitive	multiple synthesis steps
fluoride^12^	**38**	hydroquinone	silyl moieties	23.8 μM–1.19 mM	23.8 μM	water samples	5 min	CV	easy, simple to handle	solubility issues
	**39**	methoxy hydroquinone	silyl moieties	2.38 μM–0.96 mM	2.38 μM	water samples	5 min	CV	user-friendly	high detection limit
fluoride^57^	**40**	hydroquinone	silyl moieties	0–100 μM	8.3 × 10^–8^ M	urine, blood, and plasma	30 min	CV, DPV	simple and high selectivity	less sensitive, and reactivity.
Pd^59^	**41**, **43**, **45**	ferrocene	allyl trigger		<1 mg/mL		20 min	DPV	high selectivity,	complex detection steps
formaldehyde^61^	**42**	N-alkylated aminoferrocene	aza-cope trigger	0.12–1000 μM	48.2 nM	whole blood	30 min	CV, DPV	direct analysis in cloudy media	not aqueous soluble.
creatinine^61^	**42**	N-alkylated aminoferrocene	Aza-cope	3.25–200 μM	1.3 μM	saliva sample	45 min	CV, DPV	high sensitivity, selectivity	poor aqueous solubility of probe
phosphine^60^	**30**	aminoferrocene	azido benzene	0.05–5 mM	13 ppm			DPV	effective; highly sensitive, selective	poor linearity concentrations
chloramine-T^62^	**26**			0.05–100 μM	6.0 nM	pharmaceutical sample	5 min	CV, amperometry	excellent selectivity and low potential	poor sensitivity
artemisinin^63^	**26**	aminophenol		2.0–200 μM	0.8 μM	tablets	5 min	CV	less overpotential	lack of repeatability

**Figure 12:**
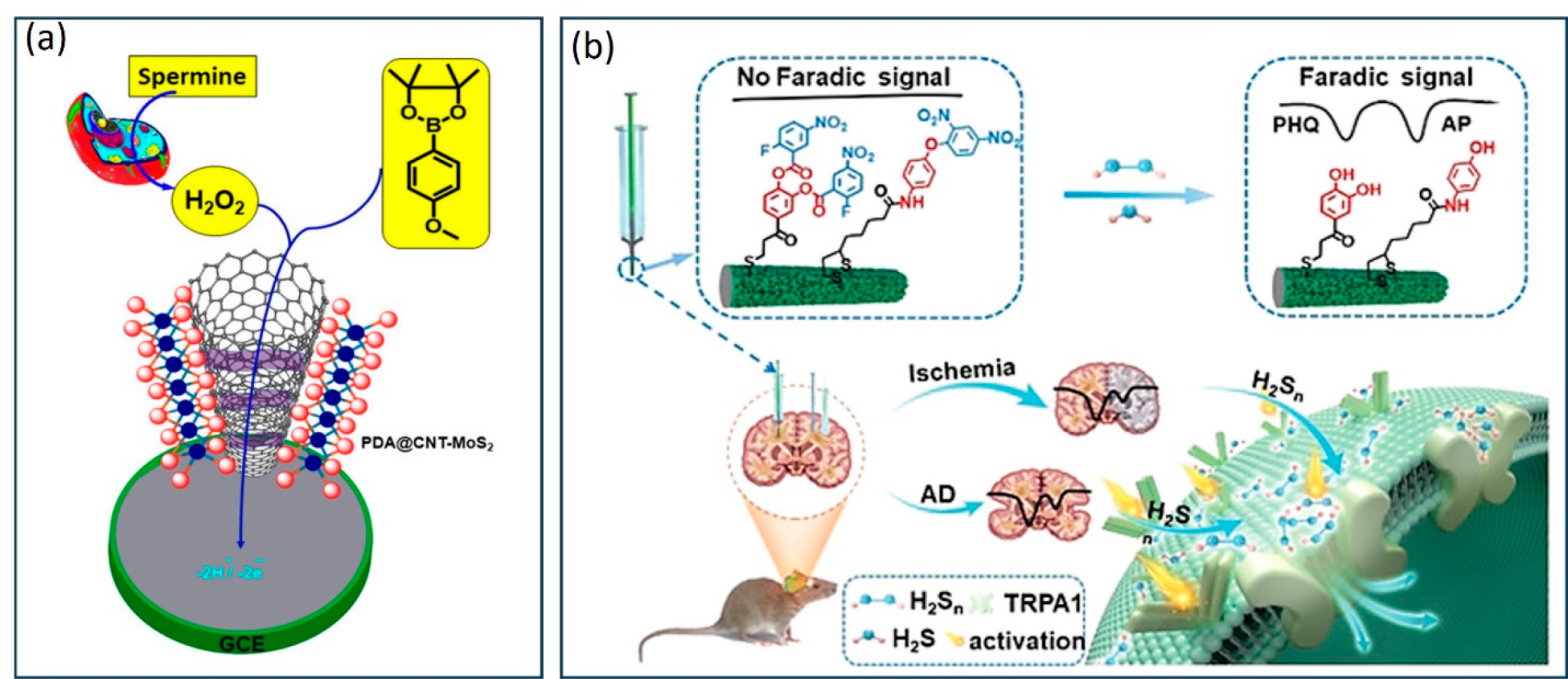
(a) Ratiometric sensing of H_2_O_2_ using substrate **25**, 4-methoxyphenylboronic acid pinacol ester, and PDA@CNT–MoS_2_ modified electrode as a signal amplifier. Reprinted from ref (48). Copyright 2019 American Chemical Society. (b) Simultaneous monitoring of endogenous H_2_S_*n*_ and H_2_S in mouse brain. Reprinted with permission from ref (53). Copyright 2019 John Wiley and Sons.

Hydrogen peroxide is an important cell-signaling molecule, involved in several physiological and pathological functions.^46^ Besides, H_2_O_2_ is the substrate for horseradish peroxidase (HRP), which is the most popular tag enzyme employed in biochemical assays.^47^ Selective determination of H_2_O_2_ among other reactive oxygen species such as hydroxyl radical (˙OH) and superoxide anion O_2_^.–^ are still challenging by traditional electrochemical sensors. Significant numbers of electrochemical redox substrates are reported for highly selective and sensitive determination of H_2_O_2_. Phenyl boronic esters are the most widely used trigger moieties in most of the H_2_O_2_ substrates because of their special affinity with H_2_O_2_.^13,14,48−50^ 4-methoxy phenol,^48^ aminoferrocene,^14^*p*-aminopheol,^49^ hydroquinone,^50^ and naphthoquinone^50^ are reporters employed. In one such work, we reported a turn-on ratiometric electrochemical substrate, **25**, 4-methoxyphenylboronic acid pinacol ester derivative (4-MPBP) for H_2_O_2_. The reporter installed in the substrate is 4-methoxy phenol, which shows excellent redox signals, originating from their quinone-hydroquinone chemistries. The sensing probe consists of 4-methoxy phenol bearing a credit unit (boronic acid pinacol ester) for targeting H_2_O_2_. The target analyte-triggered group of chemical transformation delivers a free signal reporter for 4-MP (Figure 12a).^48^ In addition, we have developed a highly sensitive modified GCE using polydopamine@carbon nanotubes-molybdenum disulfide (PDA@CNT–MoS_2_) modification. The blend of electrochemical redox substrate strategy, with electrocatalytic signal amplification technique has delivered outstanding assay performance with a detection limit of 4.1 nM and a linear range of 0.01–100 μM. Interestingly, the substrate **25** in association with PDA@CNT–MoS_2_ able to achieve real-time in vivo monitoring of the endogenously produced H_2_O_2_ in Caco-2 and MCF-7 cells through spermine–polyamine analogue and phorbol 12-myristate 13-acetate induction in SSAT/PAO gene and protein kinase C, respectively. In another interesting work, a substrate based on boronic esters linked with ferrocene was synthesized and tested for ratiometric detection of H_2_O_2_.^14^ The authors installed different linkers to identify the optimum structural criteria required to attain a selective ratiometric electrochemical sensor. Among the p-benzyl carbamate and allyl carbamate linkers, *p*-benzyl carbamate linker provided better results. Substrate **27**, *p*-benzyl carbamate linker, is demonstrated for application as a ratiometric H_2_O_2_ sensor. The authors attached nonspecific triggers such as benzyl and allyl and demonstrated that these substrates are unable to react with H_2_O_2_, such control studies are highly recommended to prove the special chemical affinity between trigger moieties and target analytes. In addition to H_2_O_2_ sensing, the authors extended the applicability of the substrate for indirect detection of glucose via an enzymatic assay. Glucose concentrations up to 50 μM can be determined with this innovative ratiometric electrochemical glucose analysis.

Xu et al. demonstrated two electrochemical substrates, *p*-aminophenylboronic acid (**26**)^49^ and *p*-hydroxyphenylboronic acid (**24**)^13^ for detecting H_2_O_2_. These two substrates use boronic acid as triggers and either *p*-aminophenol or *p*-hydroxyphenol as the reporters, and they are directly attached without bridged via a linker. In addition, *p*-hydroxyphenylboronic acid substrate is used for the indirect determination of these H_2_O_2_ sensors are used to uric acid and uricase and this sensor exhibited a linear response over the range of 1 μM–1 mM, 1–500 μM, and 0.005–0.1 U/mL with the detection limits of 0.31 μM, 0.3 μM, and 0.005 U/mL for H_2_O_2_, uric acid, and uricase, respectively. It is no surprise that H_2_O_2_ sensors can be used for the indirect determination of other analytes because H_2_O_2_ is a well-known byproduct of several biochemical processes. Naphthoquinone/naphthohydroquinone based redox-active substrate, **28** is developed for H_2_O_2_ using self-immolative boronic ester as the trigger unit.^50^ It is an autocatalytic kinetic trace reaction that shows characteristic lags and exponential signals and detects concentrations as low as 0.5 μM H_2_O_2_ and 0.5 nM naphthoquinone. Efforts are also made to attach electrochemical substrates on the electrode surface. For example, Dong et al., attached a H_2_O_2_ substrate, **46**, 5-(1,2-dithiolan-3-yl)-*N*-(4-(4,4,5,5-tetramethyl-1,3,2-dioxaborolan-2-yl)phenyl)pentanamide (BA), on a carbon fiber microelectrode (CFME) coated with Au cones.^51^ This probe BA is composed of aminophenol reporter moiety and boronic ester trigger group. The S groups attached at the end of the substrate are chemically bonded with Au cones via Au–S linkage. The substrate immobilized electrode is used for assaying H_2_O_2_, such approaches provide faster electron kinetics because the reporter units are directly attached to the electrode surface. Also, this approach eliminates the solubility issues of the substrates.

Salicylic acid is the primary metabolite of aspirin responsible for the aspirin’s pharmacological activities of aspirin. Although regular low-dose aspirin is helps prevent neurodegenerative diseases, its excessive level in the blood is toxic and sometimes lethal. Accurate determination of salicylic acid levels in blood is needed in hospitals. Although salicylic acid is electrochemically active, it is oxidation signal usually occurs at high positive potential window and its oxidized product causes electrode poisoning issues. To overcome these limitations, our group has developed a substrate **19**, SAF based competitive electrochemical assay.^42^ The substrate is composed of salicylate trigger moiety linked to an aminoferrocene reporter through a carbamate linkage. In presence of NADH at aerobic conditions, salicylate hydroxylase catalyzed the decarboxylative hydroxylation of SAF and released a redox reporter aminoferrocene. The addition of SA inhibits the disassembly of SAF by SHL because the salicylate formed by the hydrolysis of SA reacts with SHL, which has inhibited the amount of SHL. As a result, the electrochemical signal of the reporter has been decreased, allowing a competitive assay for salicylic acid. Aminoferrocene revealed is voltammetric signal at −50 mV (vs Ag|AgCl), which is more than 500 mV minimized overpotential than the signal of salicylic acid. In addition, this approach allows to develop a fouling-free salicylic acid assay.

Cysteine, homocysteine, and glutathione are biothiols associated with the regulation of physiopathological functions. In clinical settings, there is a need to develop a precise analytical tool to discriminate and quantify these three biothiols to diagnose the related pathologies. Our group developed an electrochemical substrate, **29**, ferrocene carbamate phenyl acrylate (FCPA), for the selective determination of cysteine among other biothiols. The electrochemical signal generated by FCPA is specific to cysteine but insensitive to other amino acids and structurally similar other biological species. The sulfur in cysteine undertakes a Michael addition with the acrylate unit of FCPA followed by an intramolecular ring closure with the free amine in cysteine to eject the phenol moiety of FCPA; in contrast, homocysteine, and glutathione are unable to undergo the ring closure reaction due to either the reaction being energetically unfavorable or due to the lack of a free amine.^52^ The corresponding phenol moiety of FCPA undergoes the quinone–methide rearrangement to unmask the reporter aminoferrocene. The substrate was able for real-time tracking and quantification of cellular cysteine productions in *E. coli*, along with a whole blood assay to determine levels of cysteine.

H_2_S plays vital physiological and pathological roles as a gasotransmitter. Especially, the maintenance of healthy concentration levels of H_2_S (10–600 μM) is extremely crucial for brain function. The existing ion-selective electrodes are not suitable for monitoring biological H_2_S because of the extreme alkaline pH requirements. Interferences from other gasotransmitters such as nitric oxide and carbon monoxide are also major issues. The lack of reliable approaches for real-time quantitative determination of H_2_S and polysulfides (H_2_S_n_) in living systems limits the exploration of their potential physiological and therapeutic roles in biological functions. We have developed two sets of electrochemical substrates, **30** (azido benzyl ferrocene carbamate, ABFC) and **31** (*N*-alkyl azido benzyl ferrocene carbamate, NABFC), for selective and sensitive determination of biological H_2_S.^10^ The azide trigger group specifically recognizes H_2_S and triggers the release of reporter from the substrate. The detection limits are 0.32 μM (ABFC) and 0.076 μM (NABFC). NABFC substrate performed better than ABFC because of its improved solubility. None of the other biological species reacted with ABFC and NABFC, meaning a high level of selectivity and specificity the electrochemical substrate provides for H_2_S. The substrates are successful in real-time electrochemical quantification of endogenous H_2_S in living cells.

Density functional theory (DFT) calculations and electrochemical measurements were used to design substrates **32** (3,4-bis((2-fluoro-5-nitrobenzoyl)oxy)-benzoic acid) and **33** (*N*-(4-(2,5-dinitrophenoxy) phenyl)-5-(1, 2-dithiolan-3-yl)pentanamide) for H_2_S_*n*_ and H_2_S, respectively (Figure 12b).^53^ A microelectrode decorated with mesoporous Au film was used to attach the substrates **32** and **33**, and the resultant sensor can simultaneously measure H_2_S_*n*_ from 0.2 to 50 μM and H_2_S from 0.2 to 40 μM in well-separated peak potentials. With the help of this sensor, it was found that the expression of TRPA1 protein positively correlated with the levels of H_2_S_n_ under both ischemia and Alzheimer’s disease. The same group redesigned another substrate for H_2_S_*n*_, **34**, with the installation of additional bidentate thiols for improved sensing.^54^ The installed bidentate thiols helped the substrate to attach to the Au microelectrode surface via Au–S chemistry, allowing the preparation of a stable modified electrode. The bis-electrophilic (fluorobenzoates) trigger groups of **34** were able to recognize two – SH units at H_2_S_n_ via nucleophilic aromatic substitution and trigger the ejection of pyrocatechol reporter unit, eliciting a well-defined faradic current signal at +0.24 V (vs Ag/AgCl). The H_2_S_*n*_-specific substrate attached Au microelectrode sensing system demonstrated high selectivity for real-time tracking of H_2_S_n_ in a linear range of 0.25–20 μM and employed for in vivo assaying of H_2_S_*n*_ in mouse brains with ischemia, which showed high selectivity, accuracy, and stability.

**Figure 13:**
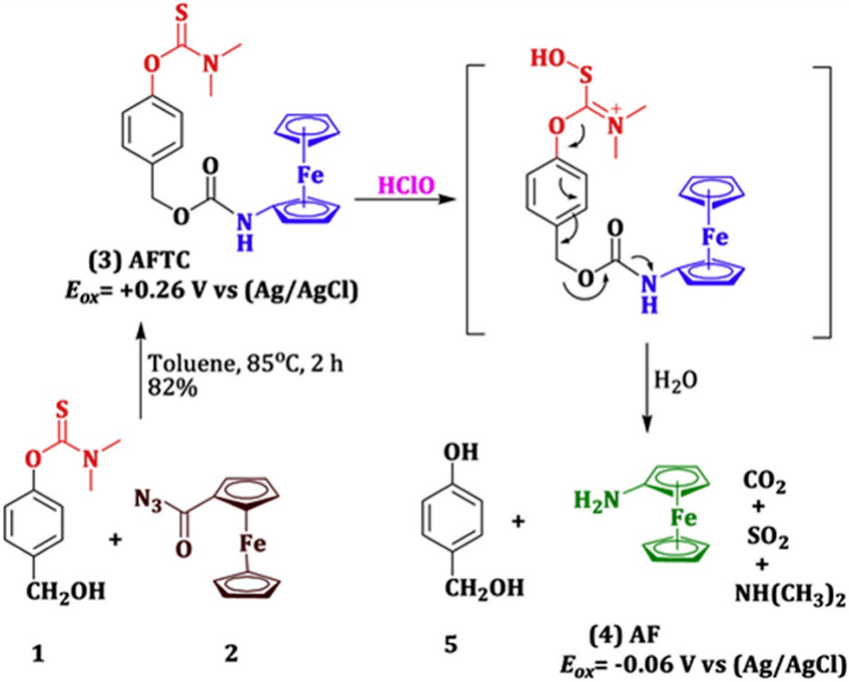
Electrochemical working principle of substrate AFTC for HClO detection. Reprinted with permission from ref (55). Copyright 2020 Elsevier.

Hypochlorous acid (HClO) is a type of reactive oxygen species, endogenously produced through a myeloperoxidase (MPO)-catalyzed reaction between H_2_O_2_ and Cl^–^ ions. Our research group recently developed an electrochemical substrate, **35**, aminoferrocene thiocarbamate (AFTC), for the specific and selective determination of HClO (Figure 13).^55^ The substrate consists of a reporter, aminoferrocene linked with a dimethylthiocarbamate trigger moiety via a hydroxyl benzyl alcohol linker. Because of the special chemical affinity between HClO and dimethylthiocarbamate, the substrate can react and undergoes a self-immolative reactions to generate the reporter molecule. The developed system delivered wide linearity and a very low LOD of about 75 nM. Real-time *in situ* quantification of HClO was executed in macrophages.

Fluoride ions (F^–^) are essential trace elements, involved in various biochemical processes, the most notorious one is its vital role in dental health. Regular intake of healthy amounts is good for health and prevents dental caries, however, excessive levels cause serious health issues. The permissible upper limit of fluorides in drinking water is 2 ppm, while the permitted range of fluoride content in toothpaste is 0.50–1.50 mg.g^–1^.^12^ The development of analytical methods is essential for the accurate determination of fluoride. Our research group developed substrates **37** (ferrocenyl carbamate derivate (FCCD),^56^**38** (1,4-Bis(*tert*-butyldimethylsiloxy)benzene, H_2_Q′)^12^ and **39** (1,4-Bis (*tert*-butyldimet hylsiloxy)-2-methoxybenzene, MH_2_Q′)^12^ for fluoride, while Tao et al., reported a substrate **40** (spiropyran-hyroquinone based substrate, SPOSi)^57^ for fluoride detections. All three substrates employed a silyl ether unit as the trigger group, because it not only provides special chemical affinity for fluoride via F–Si bond formation but also effectively masks the electrochemical signal of hydroquinone. The LODs for fluoride detection are 0.51 μM (FCCD), 23.8 μM (H_2_Q′), 2.38 μM (MH_2_Q′), and 83 nM. In the case of FCCD substrate, it undergoes a nucleophilic substitution reaction, leading to the removal of the silyl group through a 1,6-quinone-methide rearrangement with concomitant release of reporter ferrocenyl amine.^56^ In the case of H_2_Q′ and MH_2_Q′, fluoride triggered the cleavage of the Si–O bond and subsequently removed the silyl-protecting group through quinone-type rearrangement with concomitant release of reporter hydroquinone or *o*-methoxy hydroquinone.^12^ H_2_Q′ and MH_2_Q′ are electrochemically inactive, the electrochemical properties of the hydroquinone are completely masked, and only fluoride can unmask the hydroquinone allowing a highly sensitive signal. Similarly, the SPOSi substrate also depends on hydroquinone reporter, in addition, the authors used single-walled carbon nanotubes (SWCNTs) modification on the GCE to facilitate the signal amplification. The SPOSi substrate in association with SWCNTs/GCE showed nanomolar level sensitive detections compared to micromolar level detections with FCCD, H_2_Q′, and MH_2_Q′ that are not associated with nanomaterials modification. The results indicate that the use of nanomaterial electrodes is highly useful for boosting the detection limits. On the one hand, the substrate can provide a high level of selectivity, on the other hand, the nanomaterial modifications can provide signal amplification leading high level of sensitivity. The results show that using modified electrodes made of nanomaterials is a great way to increase the detection limits. High levels of selectivity can be achieved by the substrate on the one hand, and high levels of sensitivity can be achieved by the nanomaterials signal amplifications on the other hand.

Some other electrochemical substrates developed so far include **36** (ferrocene-thiourea derivative based substrate) for mercury,^58^**41** and **43**–**45** (allyl ether trigger linked ferrocene substrates) for Pd,^59^**30** (4-azido trigger linked benzyl ferrocenylcarbamate substrate) for phosphines,^60^**42** (aza-cope trigger linked aminoferrocene substrates) for formaldehyde and creatinine,^61^ and **26** (*p*-aminophenylboronic acid based substrates) for chloramine-T^62^ and artemisinin.^63^

## 5 Conclusions, Challenges, and Future Prospects

In this Review, we have summarized recent advances in the research of activity-based electrochemical substrates for chemical sensors. Special attention is given to the design strategies, sensing principles, and applications in sensing enzymes, heavy metal ions, and reactive oxygen species by highlighting several representative studies. The nature of activity-based electrochemical substrate provides advantages including simple design, operation, easy synthesis, rapid detection, immobilization-free, freedom of applicability, improved specificity of target recognition, and optimum sensitivity under complex biological medium. New concepts and sensor platforms of activity-based substrate related to the sensor performance of each biomarker sensitivity is an exciting area of sensor research to explore under potentially competing analytes. The activity based electrochemical substrate design and assembly continue to be useful for clinical, pharmaceutical, industrial, and environmental applications.

**Figure 14:**
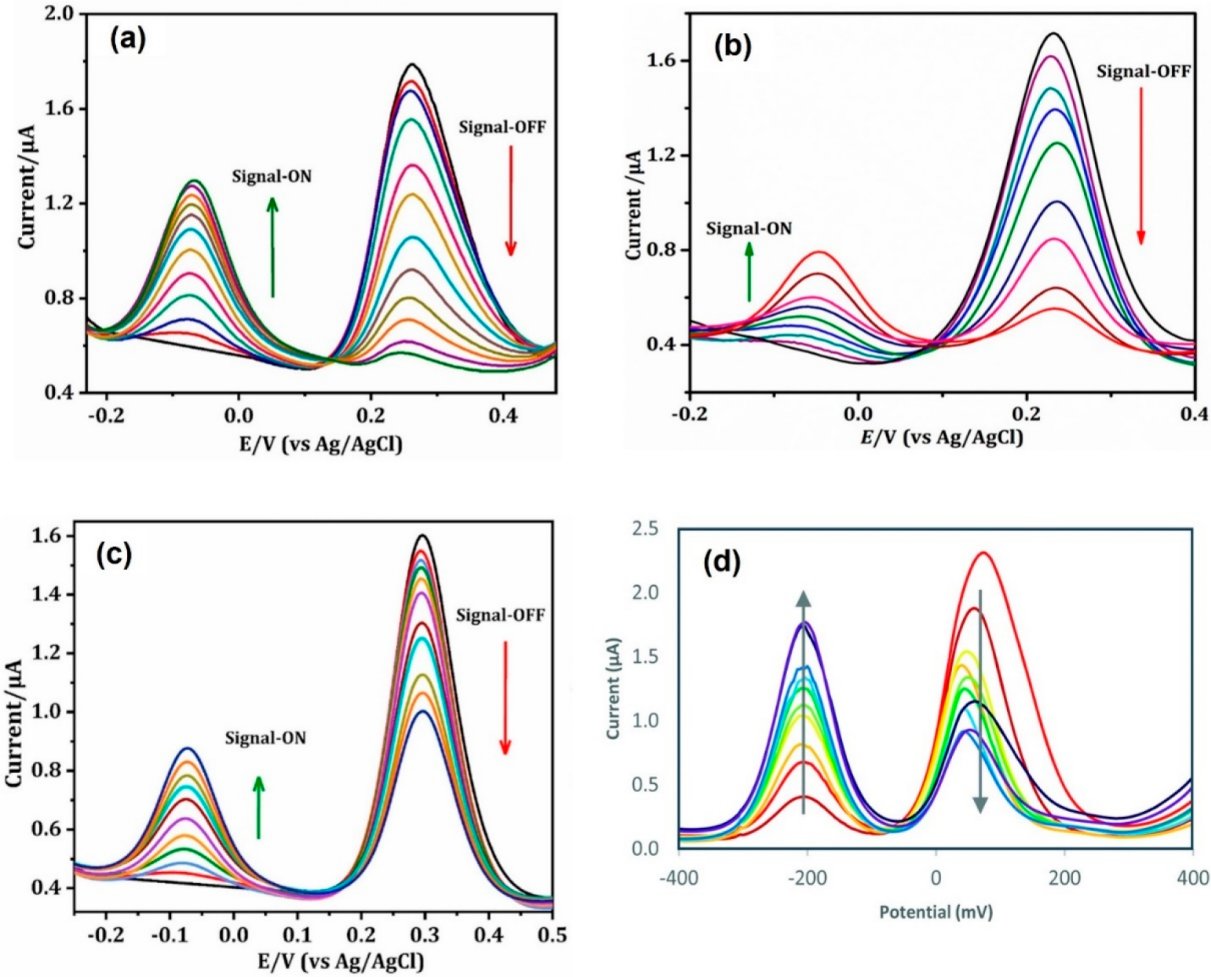
Ratiometric electrochemical sensors derived from various electrochemical redox substrates: (a) Ratiometric DPV signals of alanine-benzyl alkylated ferrocene carbamate for aminopeptidase N. The working electrode and supporting electrolyte are glassy carbon electrode (GCE) and 0.1 M PBS (pH 7.4), respectively. Reprinted with permission from ref (44). Copyright 2022 Elsevier. (b) Ratiometric DPV signals of ferrocene carbamate phenyl acrylate for cysteine. The working electrode and supporting electrolyte are reduced graphene oxide (RGO) modified GCE and 25/75% DMSO/phosphate buffer (pH 7.0), respectively. Reprinted from (52). Copyright 2018 American Chemical Society. (c) Ratiometric DPV signals of carboxyl amino ferrocene for Salmonella. The working electrode and supporting electrolyte are glassy carbon electrode (GCE) and 0.1 M PBS (pH 7.4), respectively. Reprinted with permission from ref (45). Copyright 2022 Elsevier. (d) Ratiometric DPV signals of p-benzyl carbamate ferrocenyl amine substrate for hydrogen peroxide. The working electrode and supporting electrolyte are screen-printed carbon electrodes and 50 mM pH 8.1 Tris buffer, respectively. Reprinted with permission from ref (14). Copyright 2017 Royal Society of Chemistry.

It is widely established that ratiometric sensors provide better analytical performance, especially increased accuracy compared to nonratiometric sensors. Because dual signals can be monitored in ratiometric sensors rather than just one signal in nonratiometric sensors.^64,65^ The ratio between the dual signals can be used for accurate and quantitative measurement of target analytes. In addition, ratiometric sensors are known for their greater reproducibility, accuracy and sensitivity compared to their corresponding nonratiometric sensors. Interestingly, several electrochemical redox substrates offer ratiometric sensing platforms when both substrates and reporters are capable to show signals at a distinguished potential region. In such a situation, the substrate offers switch-OFF signals (linear decrease in current signal), while the reporter offers switch-ON signals (linear increase in current signal), as shown in Figure 14. For example, alanine-benzyl alkylated ferrocene carbamate-based electrochemical substrate provides a ratiometric sensing for aminopeptidase N, at two potential points, one at +0.26 V Ag/AgCl (substrate signal) and another at 0.09 V (reporter signal),^44^ ferrocene carbamate phenyl acrylate (FCPA) based reporter provides a ratiometric sensor for cysteine at +0.25 V (substrate signal) and −0.02 V (reporter signal),^52^ carboxyl amino ferrocene based electrochemical substrate provides a ratiometric sensor for Salmonella detections at +0.30 V (substrate signal) and −0.13 V (reporter signal),^45^ and *p*-benzyl carbamate ferrocenyl amine based substrate provides a ratiometric sensor for hydrogen peroxide detections, at 0.06 V (substrate signal) and −0.20 V (reporter signal).^14^ It should be noted that a sufficient millivolts distance should be maintained between the signal zone of the reporter and probe, allowing to measure currents to draw calibration plots.

Even with the tremendous progress and efforts of numerous research groups, there are still certain drawbacks and unresolved issues with the electrochemical redox substrates. Some of them are poor sensitivity, solubility issues, probe–analyte compatibility, biofouling, and longevity. A viable electrochemical technique is still being sought for the detection of target species and disease exposure. Many strategies can be used to solve this problem but still need improvements. For example, high-sensitive reporter attachment, water-soluble units in probe and reporter fragments, nanocomposite surface functionalization on electrodes, trigger group design for probe-analyte specific interaction, nondegradable materials, etc. Another limitation is the detection mode for practical use, such as real samples, real cell culture at the electrode interface, or coculture live cell samples for high-throughput electrochemical assays. In many activity-based substrate cases, large amounts of electrolyte solution are used, which calls for large amounts of real samples for sensitive detection. There is a need to miniaturize the sampling process by optimizing its use with minimal electrolyte preparation techniques to provide a simple and miniature sensor that avoids maximum target samples and allows small volume while maintaining high sensitivity. Recently, microfluidic methodology and syringe injection have been used to precisely deliver targets to materials and device platforms with the support of electrolyte fluids. Thus, a probe extended with a microfluidic-based biosensing routine shall be prominent.

Most homogeneous electrochemical sensors focus on single-signal detection of a single target, easily leading to a false-positive diagnosis; hence, it is highly desirable to develop a multiple-signal strategy to achieve more reliable detection of the target object. Additionally, being able to detect multiple targets with multiple electrodes simultaneously improves diagnostic and treatment accuracy by allowing for more precise indications. An electrode-chip system integrated with a signal broadcast can be used as a portable sensing method to assist in understanding how biomarker levels correlate with the disease presence and progression for routine and sustained analysis of activity-based substrates which will be needed in the future. Many of the electrochemical substrates are hydrophobic, which creates solubility issues and limits the attainment of the full potential of the substrates. Making the electrochemical substrates by attaching the relevant functional groups on the substrate is essential to widely use them for practical applications. Another potential solution to this problem is immobilizing the substrates directly on the electrode surface, this resembles enzyme immobilizations to fabricate enzymatic biosensors. For this purpose, disposable electrodes such as screen-printed carbon electrodes (SPCE) are more suitable because the functionalized may damage the electrode surface if electrodes such as GCE or standard Au electrodes are used. Immobilizing electrochemical substrates on the electrode surface is one of the potential future directions that can be explored. In fact, a handful of reports successfully achieved immobilization or attachment of the substrates on the electrode surface: a H_2_S_*n*_ electrochemical probe, **34**, 4-(5-(1,2-dithiolan-3-yl)pentanamido)-1,2-phenylene bis(2-fluoro-5-nitrobenzoate),^54^ was immobilized on a Au electrode via Au–S chemistry, and a H_2_O_2_ substrate, **46**, 5-(1,2-dithiolan-3-yl)-N-(4-(4,4,5,5-tetramethyl-1,3,2-dioxaborolan-2-yl)phenyl)pent-anamide,^51^ was attached on a carbon fiber microelectrode coated with Au cones via Au–S chemistry.

Still, most of the reports use conventional electrodes such as GCE. This is because the research groups are generally organic chemists who are more focused on synthesizing substrates and testing their biosensing properties with target analytes. However, the clinical assay is moving toward more PoC assay set up with miniaturized chip-based assays. The use of SPCE or similar disposable electrodes should be integrated with electrochemical redox substrates to translate this method into more practical applications. Multidisciplinary research collaborations are highly recommended, among organic chemists, electrochemists, analytical chemists, and electrical engineering to translate the electrochemical substrates into a miniaturized device, such as disposable glucose strips.

Some of the reports successfully integrated electrochemical substrates with commercial glucose sensors, which are potential approaches for practical applications. Such approaches use glucose as the signal reporter, which is not a preferable mediator compared to mediators such as hydroquinone and ferrocene. It should be noted that the working range of the commercial glucose meters is in the millimolar range. However, commercial glucose meters with disposable test strips are highly reliable, ubiquitous, user-friendly, and inexpensive. In fact, the commercial glucose sensors market is the most active one in the biosensors market arena. Future directions could be focused more on developing glucose based electrochemical substrates. With the use of continuous glucose monitoring devices (CGM) coupled with electrochemical redox reporters, further advancement can be made for continuous real-time sensing applications, which are getting more attention for precision medicine. Although, the common redox reporters can reveal sensitive electrochemical signals, modifying the electrode surface with nanomaterial proved to be a versatile approach to increase sensitivity. Such modified electrodes are designed to catalyze the redox reactions of the reporters. In such cases, the electrochemical redox substrates provide the needed selectivity and sensitivity, while the nanomaterials amplify the electrochemical signals. Such integration is effective for boosting sensitivity and selectivity and for monitoring practical samples. For instance, a SWCNT modified GCE was used to facilitate the signal amplification of the fluoride sensing substrate.

Several substrates require 1 h of incubation time with target analytes, which makes the analysis time longer. One possible route to minimize the analysis time is to focus on optimizing linkers. In fact, often, most efforts are focused on designing triggers and reporters with little effort are focused on designing effective linkers. A considerable amount of optimization should be done to find effective linkers for achieving faster elimination kinetics, which is essential to minimize the analysis time. Future directions could be taken on immobilizing or chemically attaching the substrates on the electrode surface. Such attachments provide faster electrode kinetics because the reporters are directly attached to the electrode surface.^51^ Unlike being limited by diffusion when reporters are released in electrolytes, the electrode kinetics is surface confined when reporters are attached to the electrode surface.
